# Dual Role of CRABP2 in Colorectal Cancer: Oncogenesis via Nuclear RB1 and Cytoplasmic AFG3L2/SLC25A39 Axis, While Limiting Liver Metastasis through Cytoplasmic AFG3L2/PINK1/Parkin‐Mediated Mitophagy

**DOI:** 10.1002/advs.202500552

**Published:** 2025-04-30

**Authors:** Chuanxin Tian, Sheng Yang, Chuan Zhang, Renzhong Zhu, Chen Chen, Xiaowei Wang, Dongsheng Zhang, Qingyang Sun, Hengjie Xu, Hongxu Nie, Yue Zhang, Dongjian Ji, Junwei Tang, Kangpeng Jin, Yueming Sun

**Affiliations:** ^1^ Department of General Surgery, Colorectal Institute of Nanjing Medical University The First Affiliated Hospital of Nanjing Medical University Nanjing 210029 China; ^2^ Jiangsu Province Engineering Research Center of Colorectal Cancer Precision Medicine and Translational Medicine Nanjing 210029 China; ^3^ Collaborative Innovation Center for Cancer Personalized Medicine Nanjing Medical University Nanjing 210029 China; ^4^ Institute of Translational Medicine Medical College Yangzhou University No.136 Jiangyang Road Yangzhou 210029 China

**Keywords:** AFG3L2, cellular retinoic acid–binding proteins, colorectal cancer, CRABP2, liver metastasis, mitophagy, PINK1, RB1

## Abstract

Colorectal cancer (CRC) progression and metastasis involve numerous regulatory factors. Among these, cellular retinoic acid‐binding protein 2 (CRABP2) has been implicated as both a tumor activator and suppressor. Here, it is aimed to clarify the role of CRABP2 in CRC growth and metastasis and explore the underlying molecular mechanisms mediating its cellular functions. Using both in vitro and in vivo models, including a colonocyte‐specific CRABP2 conditional knockout mouse model (*Crabp2* ^ΔIEC^) and a subcutaneous tumorigenesis assay in BALB/c nude mice, it is shown that nuclear CRABP2 enhances tumor growth by interacting with and downregulating the tumor suppressor RB1, whereas cytoplasmic CRABP2 suppresses CRC liver metastasis by interacting with AFG3L2 and promoting mitophagy. In addition, the AFG3L2–SLC25A39 axis is identified as a distinct mechanism by which cytoplasmic CRABP2 increases mitochondrial glutathione stability to promote cell proliferation independent of the nuclear RB1 pathway. Notably, analysis of tissue from CRC patients reveals that CRABP2 protein has distinct prognostic implications and functional roles in the progression and metastasis of CRC dependent on its subcellular localization. Ultimately, by elucidating the role of CRABP2 in CRC, it is aimed to provide new insight into disease pathogenesis and inform the development of therapeutic interventions.

## Graphical abstract

CRABP2 plays a dual role in colorectal cancer: it promotes proliferation and suppresses apoptosis through the nuclear CRABP2/RB1 axis and the cytoplasmic AFG3L2/SLC25A39 axis. Meanwhile, it inhibits colorectal liver metastasis via AFG3L2/PINK1/Parkin‐mediated mitophagy. These findings highlight the multifaceted role of CRABP2 in CRC, offering new insights into disease pathogenesis and potential therapeutic interventions.

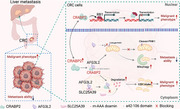

## 1 Introduction

As the third most common malignancy and the second leading cause of cancer‐related deaths globally, colorectal cancer (CRC) ranks highly among the leading causes of cancer incidence and mortality worldwide.^[^ ^1^ ^]^ The poor prognosis and elevated mortality rates among CRC patients primarily result from distant metastasis, particularly to the liver, which, due to its unique blood supply, is the most common metastatic site, with approximately half of all CRC patients developing colorectal liver metastasis (CRLM).^[^ ^2^ ^]^ Critically, despite the availability of various treatment modalities, the prognosis for patients with CRLM remains grim, representing a significant clinical challenge.^[^ ^3^ ^]^ Thus, further studies exploring the underlying mechanisms of CRLM are urgently needed to identify therapeutic targets and facilitate the development of more effective treatment strategies.

Mitophagy—the selective degradation of dysfunctional mitochondria—is an important mechanism for maintaining mitochondrial homeostasis and cellular equilibrium and has been closely linked to cancer, immunity, and tissue loss.^[^ ^4^ ^]^ This process is regulated by factors that include Parkin and phosphatase and tensin homolog (PTEN)‐induced putative kinase 1 (PINK1), a critical regulator of mitophagy that is associated with various diseases, including tumors.^[^ ^5^ ^]^ In colon tumors, PINK1 was shown to inhibit tumor growth by activating p53, thereby influencing metabolic reprogramming.^[^ ^5^, ^6^ ^]^ Mitophagy dysfunction can further promote tumorigenesis via the accumulation of protein damage and modulation of the tumor microenvironment.^[^ ^7^ ^]^ In addition, mitophagy enhances CD8^+^ T cell antitumor activity through antigen processing/presentation and regulates cancer cell survival via metabolic reprogramming‐induced oxidative stress control.^[^ ^8^ ^]^These multifunctional roles for mitophagy in apoptosis, autophagy, and antioxidant pathways suggest the therapeutic potential of targeting this pathway across tumor initiation, progression, and drug resistance.^[^ ^9^ ^]^

Cellular retinoic acid–binding proteins (CRBPs), including CRBP1, CRBP2, CRABP1, CRABP2, and fatty acid–binding protein 5 (FABP5), function to transport and protect retinol and its metabolic products.^[^ ^10^ ^]^ These proteins also interact with related enzymes and nuclear receptors to facilitate the efficient utilization of retinol metabolites and facilitate their metabolism.^[^ ^11^ ^]^ Among the CRBPs, CRABP1 and CRABP2 modulate cellular metabolism and molecular interactions by binding to retinol and related compounds, thereby influencing processes such as proliferation and differentiation.^[^ ^12^ ^]^ In particular, CRABP2, a cytoplasmic‐to‐nuclear transport protein that regulates gene expression and enhances RNA stability, was found to be closely linked to the progression, metastasis, drug resistance, and immune regulation in various cancers. CRABP2 exerts these effects by modulating diverse biological processes in tumor cells, including proliferation, apoptosis, invasion, migration, and angiogenesis. Accordingly, recent studies have reported abnormal expression patterns of CRABP2 in multiple tumors, with expression levels strongly correlating with cancer prognosis. However, the mechanisms of action for CRABP2 are complex, and the protein can act as both a tumor suppressor and a promoter.^[^ ^11^, ^12^, ^13^ ^]^

In the present study, we investigated the role of CRABP2 in CRC progression. Our findings highlight complex mechanisms of action for CRABP2 in CRC, revealing dual functions in aggravating CRC progression while limiting CRLM. Moreover, the subcellular localization of CRABP2 appears to have distinct prognostic value for predicting the progression and metastasis of CRC. Ultimately, by elucidating the complex roles of CRABP2 in CRC progression, we aim to uncover valuable insights into disease pathogenesis and guide therapeutic strategies.

## 2 Results

### 2.1 CRABP2 Promotes Tumor Growth by Enhancing Proliferation and Suppressing Apoptosis

We first compared the expression of five CRBPs—retinol‐binding protein 1 and 2 (RBP1/2), CRABP1, CRABP2, and FABP5—in normal (N) versus tumor (T) tissues in The Cancer Genome Atlas (TCGA)‐Colon Adenocarcinoma (COAD) dataset. Results show that only RBP2 and CRABP2 were significantly upregulated in tumors relative to normal tissue (Figure S1A, Supporting Information), with CRABP2 showing the most pronounced upregulation *(p* < 0.001). We further validated the significant differential expression of CRABP2 in tumor versus normal tissues in additional datasets (i.e., GSE87211, GSE20842, and GSE41258), all of which showed upregulation in tumor tissue with *p*‐values <0.0001 (Figure S1B, Supporting Information).

**Figure 1:**
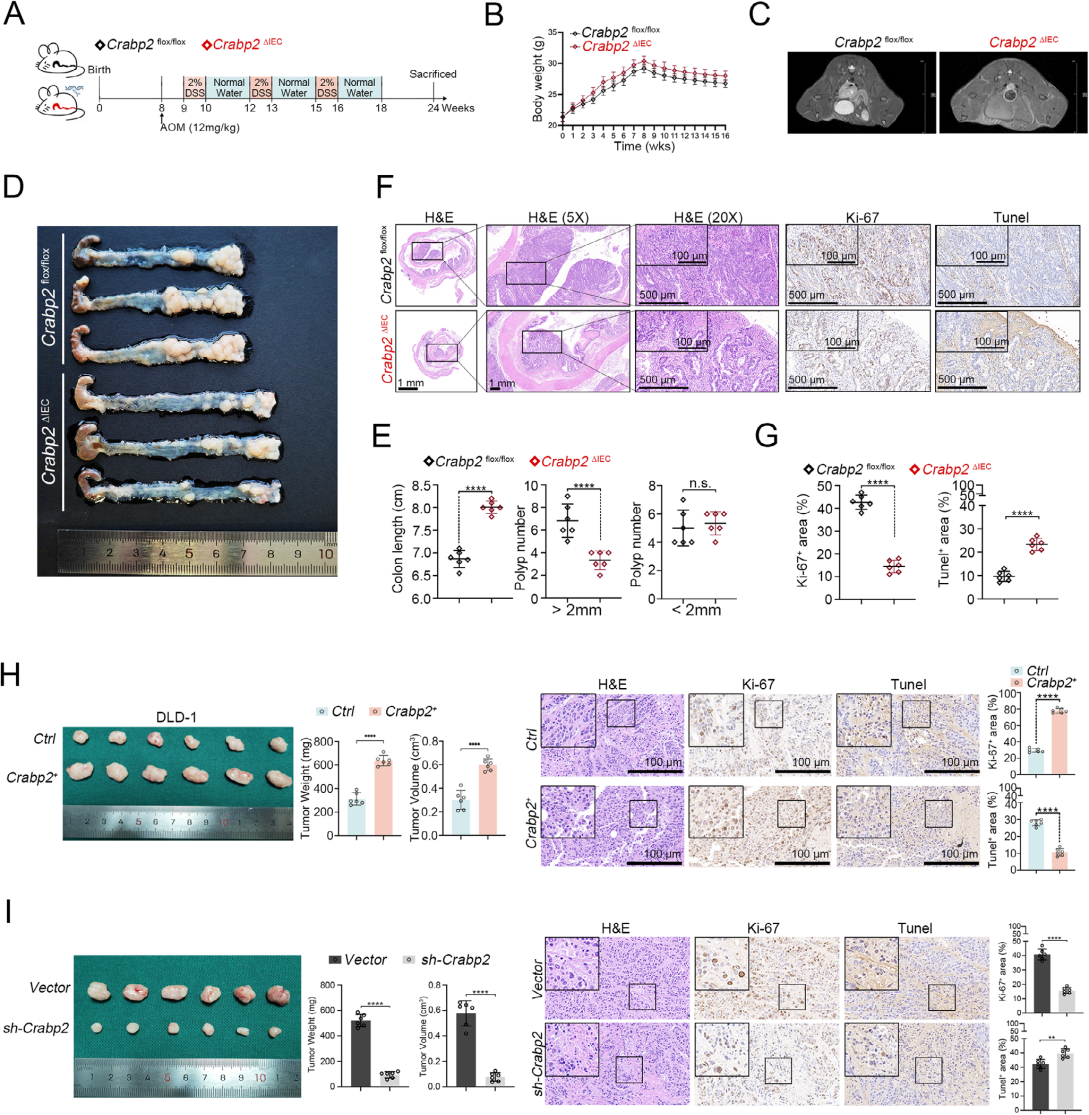
CRABP2 promotes tumor growth by enhancing proliferation and suppressing apoptosis. A) Colonocyte‐specific *Crabp2*‐knockout (*Crabp2* ^ΔIEC^) and *Crabp2* ^flox/flox^ control mice were analyzed in an AOM‐DSS‐induced model of CRC. B,C) Body weight (B) and MRI analysis (C) of *Crabp2* ^ΔIEC^ and *Crabp2* ^flox/flox^ mice with AOM‐DSS–induced CRC (*n* = 6). D,E) Colon length (D) and number of tumors per colon in *Crabp2* ^ΔIEC^ and *Crabp2* ^flox/flox^ mice with AOM‐DSS–induced CRC (*n* = 6). F,G) H&E staining, immunohistochemical staining for Ki‐67, and TUNEL staining of the tumors shown in (D). H,I) Subcutaneous tumorigenesis assays in nude mice injected with CRABP2‐overexpressing (*CRABP2* ^+^) or control (*Ctrl*) cells (H) or with shRNA‐mediated CRABP2‐knockdown (*sh‐CRABP2*) or vector control (*Vector*) cells (I). Tumor weight and volume, H&E staining, immunohistochemical staining for Ki‐67, and TUNEL staining are shown (*n* = 6). Original magnification ×1, ×5 (F), scale bar = 1 mm; ×20 (F), scale bar = 500 µm; ×40 (F, H, I), scale bar = 100 µm. The data are presented as the mean ± SD. n.s. = not significant, ****p < 0.0001, as analyzed by *t*‐test (E, G, H, I).

To investigate the role of CRABP2 in CRC progression, we next generated a conditional knockout mouse model specifically targeting CRABP2 in colonocytes (*Crabp2* ^ΔIEC^; Figure S1C–G, Supporting Information) and subsequently established an azoxymethane and dextran sodium sulfate (AOM‐DSS)–induced^[^ ^6^ ^]^ model of CRC in these animals (**Figure** **1**A). We found that the *Crabp2* ^ΔIEC^ group experienced less weight loss than *Crabp2* ^flox/flox^ control mice, with CRC stages confirmed by magnetic resonance imaging (MRI; Figure 1B,C). Notably, the *Crabp2* ^ΔIEC^ group also formed smaller tumors and exhibited longer colon lengths than control mice (Figure 1D,E; Table S1, Supporting Information). Immunohistochemical analysis further revealed a decreased level of Ki‐67 staining and an increased level of terminal deoxynucleotidyl transferase dUTP nick‐end labeling (TUNEL) staining in the *Crabp2* ^ΔIEC^ compared with the control group (Figure 1F,G; Table S1, Supporting Information).

We then established stable CRABP2 overexpression and short‐hairpin (sh)RNA‐mediated CRABP2‐knockdown cell lines (Figure S1H,I, Supporting Information) and used these cells to establish subcutaneous tumors in BALB/c nude mice.^[^ ^14^ ^]^ Results show that CRABP2 overexpression enhanced tumor growth relative to control cells, leading to increased levels of Ki‐67 staining and decreased levels of TUNEL staining (Figure 1H), with CRABP2 knockdown having the opposite effect (Figure 1I). We further validated these results by performing in vitro experiments (Figure S2A–D, Supporting Information). Overall, the above findings suggest that CRABP2 promotes CRC progression by enhancing tumor cell proliferation and inhibiting apoptosis.

### 2.2 CRABP2 Promotes CRC Progression by Downregulating RB Transcriptional Corepressor 1 (RB1)

**Figure 2:**
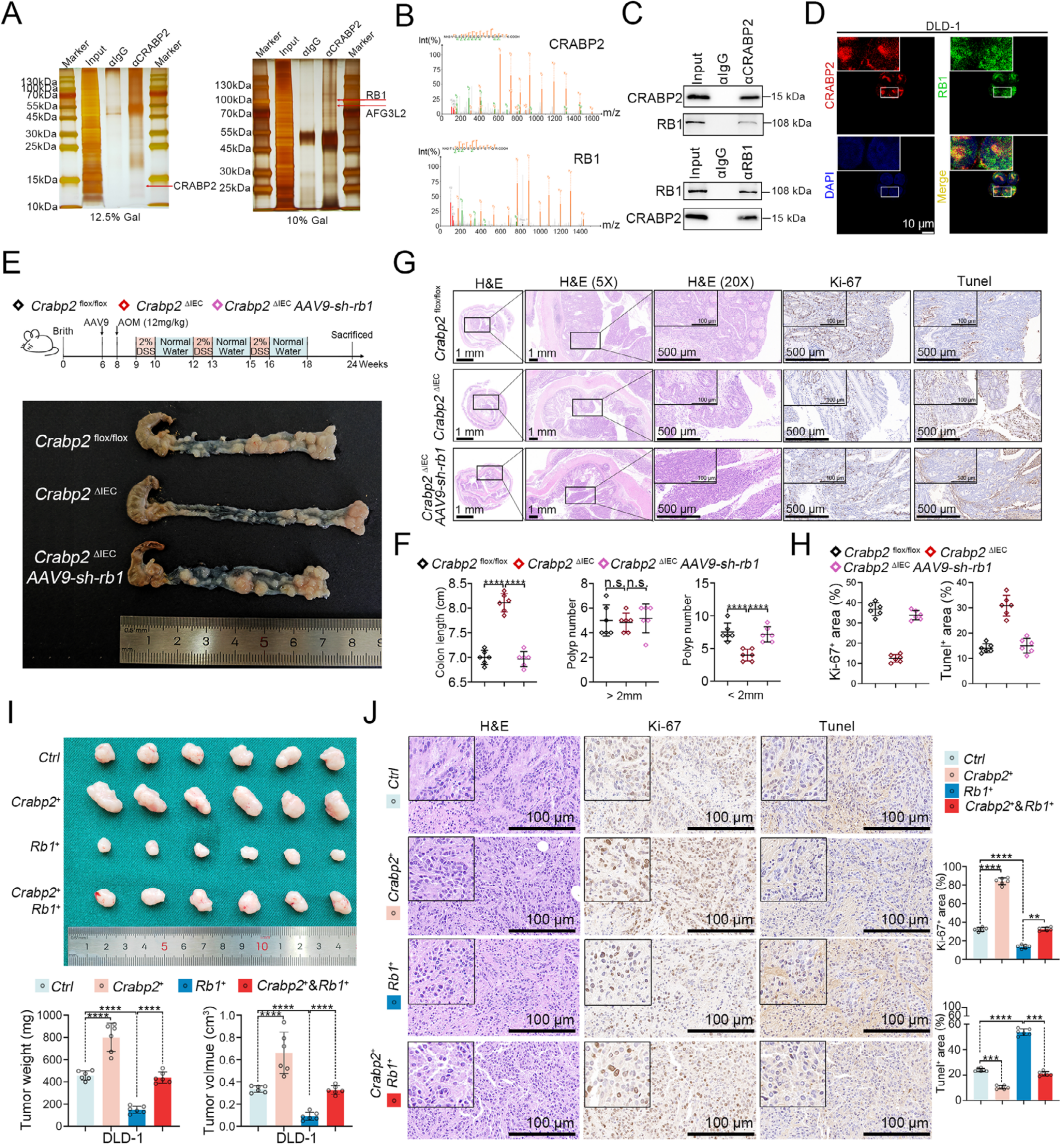
CRABP2 promotes CRC progression by downregulating RB1. A,B) Analysis of proteins interacting with CRABP2 in DLD‐1 cells by IP and MS (A) identifies the tumor suppressor RB1 (B) as a potential binding partner. C) Co‐IP experiments validating the interaction between CRABP2 and RB1 in DLD‐1 cells. D) Immunofluorescence staining for CRABP2 (red) and RB1 (green) in DLD‐1 cells. E,F) Colon length (E) and number of tumors per colon (F) in *Crabp2* ^ΔIEC^, *Crabp2* ^flox/flox^, and RB1‐knockdown *Crabp2* ^ΔIEC^ mice injected via the tail vein with AAV9 expressing shRNA targeting RB1 (AAV9‐sh‐rb1) in an AOM‐DSS–induced model of CRC (*n* = 6). G,H) H&E staining, immunohistochemical staining for Ki‐67, and TUNEL staining of the tumors shown in (E). I,J) Subcutaneous tumorigenesis assays in nude mice injected with CRABP2‐overexpressing (*CRABP2^+^*), control (*Ctrl*), RB1‐overexpressing (*RB1* ^+^), or *CRABP2^+^/RB1* ^+^ cells. Tumor weight and volume, H&E staining, immunohistochemical staining for Ki‐67, and TUNEL staining are shown (*n* = 6). Original magnification ×1, ×5 (G), scale bar = 1 mm; ×20 (G), scale bar = 500 µm; ×40 (G, J), scale bar = 100 µm; ×63 (D), scale bar = 10 µm. The data are presented as the mean ± standard deviation. n.s. = not significant, ***p < 0.001, ****p < 0.0001, as analyzed by ANOVA with Tukey's honestly significant difference test (F, H, I, J).

We next explored the molecular mechanisms by which CRABP2 promotes CRC progression by performing immunoprecipitation followed by mass spectrometry to identify proteins that interact with CRABP2 in DLD‐1 cells (**Figure** **2**A). Of the identified proteins, we focused on the retinoblastoma susceptibility gene *RB1* as an interesting target (Figure 2B). As a critical tumor suppressor, the inactivation or suppression of RB1 promotes cell division and proliferation, contributing to the development of various cancers.^[^ ^15^ ^]^ We subsequently validated the interaction between CRABP2 and RB1 in co‐immunoprecipitation assays (Figure 2C; Figure S2E, Supporting Information) and performed immunofluorescence and immunoblotting analysis, revealing that CRABP2 and RB1 co‐localize within the cell nucleus (Figure 2D; Figure S2F,G, Supporting Information).

To further investigate the roles of CRABP2 and RB1 in CRC progression, we performed adeno‐associated virus 9 (AAV9)‐mediated^[^ ^16^ ^]^ knockdown of RB1 in *Crabp2* ^ΔIEC^ mice, followed by the establishment of the AOM‐DSS–induced CRC model (Figure S2H, Supporting Information). We found that RB1 knockdown could reverse the pro‐tumorigenic effects of CRABP2, including the enhanced proliferation and suppression of apoptosis observed in *Crabp2* ^flox/flox^ relative to *Crabp2* ^ΔIEC^ mice (Figure 2E–H; Table S2, Supporting Information). Similar results were obtained in the subcutaneous tumorigenesis model with CRABP2‐ and RB1‐overexpressing cells and from in vitro experiments (Figure 2I,J; Figure S2I–K, Supporting Information). Together, these observations suggest that CRABP2 promotes CRC progression and cell proliferation and suppresses apoptosis in vitro by interacting with and downregulating with RB1 in the nucleus.

### 2.3 Cytoplasmic CRABP2 Inhibits CRC Liver Metastasis by Regulating AFG3L2

**Figure 3:**
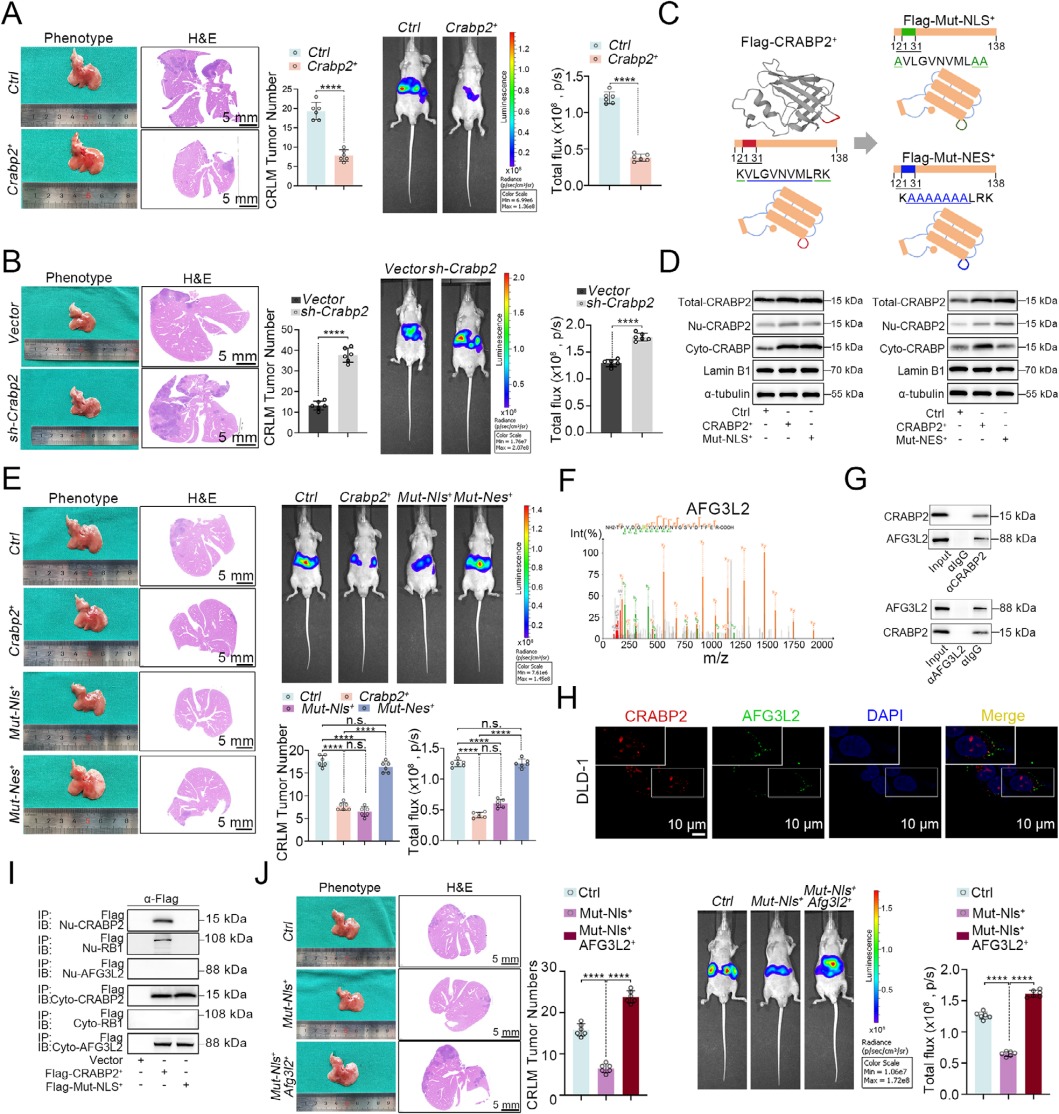
Cytoplasmic CRABP2 suppresses CRLM by interacting with AFG3L2 to maintain mitophagy. A,B) Phenotype and H&E staining of liver tissue from animals injected with CRABP2‐overexpressing (*CRABP2* ^+^) or control (*Ctrl*) cells (A) or with CRABP2‐knockdown (*sh‐CRABP2*) or control (*Ctrl*) cells (B) in a mouse model of CRLM. Quantification of CRLM and related luminescence intensities in each group are shown (*n*  =  6). C) Schematic showing the constructs directing cotranslational expression of WT CRABP2 (*CRABP2^+^*), NLS‐mutant CRABP2 (*CRABP2‐Mut‐NLS^+^*), and NES‐mutant CRABP2 (*CRABP2‐Mut‐NES^+^)* proteins. D) Immunoblot analysis measuring the levels of total CRABP2, nuclear CRABP2, cytoplasmic CRABP2, Lamin B1, and α‐tubulin in cells expressing the constructs described in (C). E) Phenotype and H&E staining of liver tissue from mice injected with *CRABP2* ^+^, *CRABP2* ^+^‐*Mut‐NLS* ^+^, *CRABP2* ^+^‐*Mut‐NES^+^*, or control (*Ctrl*) cells in a model of CRLM. Quantification of CRLM and related luminescence intensities are shown (*n*  =  6). F) Identification of AFG3L2 binding to CRABP2 in DLD‐1 cells by IP and MS. G) Co‐IP experiments validating the interaction between CRABP2 and AFG3L2 in DLD‐1 cells. H) Immunofluorescence staining for CRABP2 (red) and AFG3L2 (green). I) Interactions between CRABP2, RB1, and AFG3L2 in the nucleus and in the cytoplasm of DLD‐1 cells overexpressing CRABP2 and CRABP2‐Mut‐NLS. J) Phenotype and H&E staining of liver tissue from mice injected with *CRABP2‐Mut‐NLS* ^+^/*AFG3L2* ^+^, *CRABP2‐Mut‐NLS* ^+^, or control (*Ctrl*) cells in a model of CRLM. Quantification of CRLM and related luminescence intensities are shown (*n*  =  6). Original magnification ×1 (A, B, E, J); scale bar = 5 mm, ×63 (H), scale bar = 10 µm. The data are presented as the mean ± standard deviation. n.s. = not significant, ****p < 0.0001, as analyzed by *t*‐test (A, B) and by ANOVA with Tukey's HSD test (E, J).

Based on the above findings, we next established a CRLM mouse model^[^ ^14^ ^]^ to investigate the role of CRABP2 in CRC metastasis to the liver. Interestingly, we found that overexpression of CRABP2 significantly reduced the number of liver metastases and decreased fluorescence intensity in the liver (**Figure** **3**A), whereas CRABP2 knockdown had the opposite effect (Figure 3B). Consistent with these findings, overexpression of CRABP2 reduced the migration and invasion capabilities of CRC cells in vitro, with the opposite effect observed in CRABP2‐knockdown cells (Figure S3A–D, Supporting Information). Collectively, these results indicate that CRABP2 can inhibit CRLM, which contrasts with the pro‐tumorigenic role of CRABP2 observed in our prior experiments.

A recent study found that CRABP2 functions as a cytoplasm‐to‐nucleus shuttling protein, likely exerting distinct effects in different cellular compartments through its distinct nuclear localization signal (NLS) and nuclear export signal (NES) domains.^[^ ^17^ ^]^ Based on these findings, to amplify and test the cytoplasmic and nuclear functions of CARBP2, we generated two CRABP2‐overexpression constructs, one of which expresses an NLS‐mutant (*CRABP2‐Mut‐NLS* ^+^) that blocks nuclear translocation and accumulates in the cytoplasm and the other expressing an NES‐mutant (*CRABP2‐Mut‐NES^+^*) that blocks cytoplasmic translocation and accumulates in the nucleus (Figure 3C). Nuclear and cytoplasmic fractionation confirmed the accumulation of CRABP2 and the two variants in the expected compartments (Figure 3D). Furthermore, in the CRLM mouse model, cells expressing the *CRABP2* ^+^ or *CRABP2* ^+^‐*Mut‐NLS* ^+^ constructs showed similar suppression of liver metastases and fluorescence intensity relative to *vector* control, whereas no change versus control was observed in the *CRABP2* ^+^‐*Mut‐NES* ^+^ group (Figure 3E). Consistent results were also observed in vitro (Figure S3E,F, Supporting Information). These findings suggest that CRABP2 suppresses CRLM independent of its nuclear function.

To further explore the molecular mechanisms by which cytoplasmic CRABP2 suppresses CRLM, we reviewed the CRABP2 binding partners identified from IP and MS experiments shown in Figure 2A and focused next on the mitochondrial inner membrane m‐AAA protease component AFG3L2 (Figure 3F). This protein plays a crucial role in mitochondrial quality control, particularly in the regulation of electron transport chain complexes and maintenance of mitochondrial homeostasis.^[^ ^18^ ^]^ Subsequent co‐IP experiments revealed that CRABP2 interacts with AFG3L2 in the cytoplasm, with immunofluorescence assays further confirming the co‐localization of CRABP2 and AFG3L2 in the cytoplasm (Figure 3G–I; Figure S3G,H, Supporting Information). Moreover, in the CRLM model, we found that overexpression of AFG3L2 could reverse the inhibitory effect of CRABP2‐Mut‐NLS overexpression on CRLM in vivo (Figure 3J). Similar results were also obtained from in vitro experiments (Figure S3I,J, Supporting Information). Thus, our findings suggest that CRABP2 inhibits CRLM by downregulating AFG3L2 in the cytoplasm.

### 2.4 Cytoplasmic CRABP2 Suppresses CRLM by Interacting with AFG3L2 to Maintain Mitophagy

**Figure 4:**
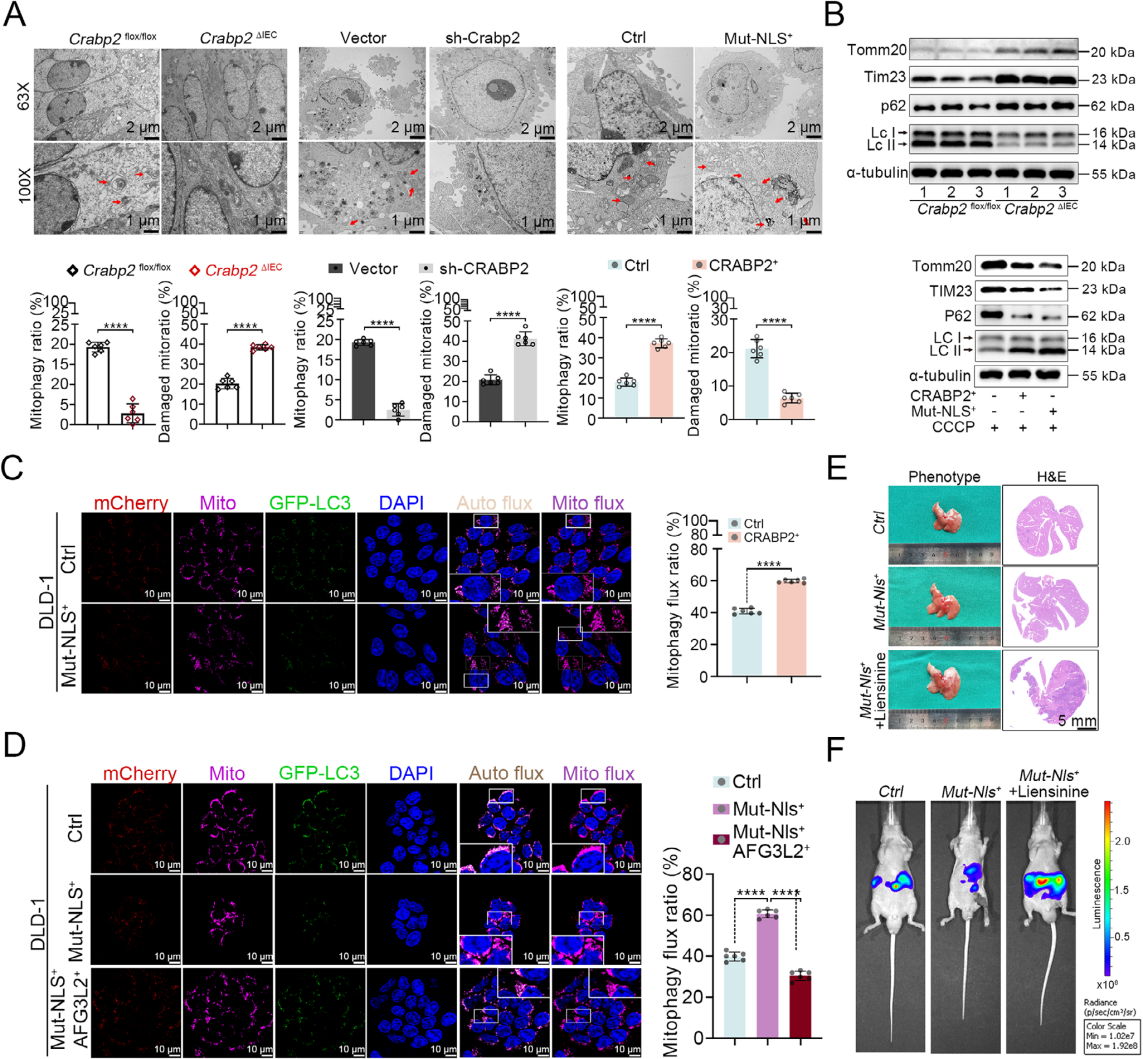
Cytoplasmic CRABP2 suppresses CRLM by interacting with AFG3L2 to enhance PINK1‐mediated mitophagy. A) TEM of colonocytes from *Crabp2* ^ΔIEC^ and *Crabp2* ^flox/flox^ mice with AOM‐DSS‐induced CRC (model shown in Figure 1A) and of CRC cells overexpressing CRABP2 or with CRABP2 knockdown; mitophagy is marked by arrows. B) Immunoblot analysis measuring expression of mitochondrial and autophagy markers and α‐tubulin in colonocytes from *Crabp2* ^ΔIEC^ and *Crabp2* ^flox/flox^ mice with AOM‐DSS‐induced CRC (model shown in Figure 1A) and in DLD‐1 cells overexpressing CRABP2 or CRABP2‐Mut‐NLS. C,D) Immunofluorescence‐based assay measuring mitophagic flux in *CRABP2‐Mut‐NLS^+^* and control (*Ctrl*) cells (C) and in *CRABP2‐Mut‐NLS^+^*, *CRABP2‐Mut‐NLS^+^/AFG3L2^+^*, and *Ctrl* cells (D). Yellow fluorescence indicates LC3 association with the autophagosome, and red fluorescence indicates LC3 association with the autolysosome. E,F) Phenotype and H&E staining of liver tissue from mice injected with *CRABP2‐Mut‐NLS^+^* cells with or without liensinine feeding or *Ctrl* cells in a model of CRLM. Quantification of CRLM and related luminescence intensities are shown (*n*  =  6). Original magnification ×1 (E), scale bar = 5 mm; ×63 (C, D), scale bar = 10 µm; ×63 (A, TEM), scale bar = 2 µm; ×100 (A, TEM), scale bar = 1 µm. The data are presented as the mean ± SD. n.s. = not significant, ****p < 0.0001, as analyzed by *t*‐test (A, C) for comparisons between two groups and by ANOVA with Tukey's HSD test (D).

A prior study reported that silencing of AFG3L2 leads to the accumulation of PINK1 on the mitochondrial surface and Parkin recruitment, resulting in mitophagy induction.^[^ ^19^ ^]^ We, therefore, performed transmission electron microscopy (TEM) to analyze the mitochondria in colonocytes from the AOM‐DSS‐induced CRC mouse model (Figure 1A) and in CRC cell lines overexpressing or knocked down for CRABP2. Results showed that CRABP2 deletion or knockdown led to the accumulation of swollen mitochondria with abnormal cristae structures, whereas CRABP2 overexpression resulted in an increased number of autophagic mitochondria (**Figure** **4**A). In addition, we performed immunoblot analysis on colonic epithelial cells isolated from AOM‐DSS–induced CRC model mice (Figure 1A) and cell lines treated with the protonophore carbonyl cyanide m‐chlorophenyl hydrazine (CCCP).^[^ ^20^ ^]^ We found that CRABP2 deletion caused the accumulation of mitochondrial proteins (TOMM20, TIM23) and p62, with a decreased LC3‐II/I ratio, whereas CRABP2 overexpression had the opposite effect (Figure 4B; Figure S4A, Supporting Information). Immunofluorescence further revealed that mitophagy flux was significantly elevated in CRABP2‐Mut‐NLS‐overexpressing cells and low in CRABP2‐knockdown cells (Figure 4C; Figure S4B, Supporting Information).

We then measured mitophagic flux in cells overexpressing CRABP2 plus AFG3L2 to clarify how these factors interact to regulate mitophagy induction. Results showed that AFG3L2 overexpression reversed the enhanced mitophagy induced by CRABP2‐Mut‐NLS overexpression (Figure 4D). In addition, we administered liensinine, an inhibitor of mitophagy,^[^ ^20^ ^]^ in the CRLM model to investigate the relationships among CRABP2, mitophagy, and CRLM. We found that liensinine blocked the inhibitory effect of CRABP2‐Mut‐NLS overexpression on CRLM in vivo (Figure 4E,F; Figure S4C, Supporting Information), with consistent results obtained from in vitro experiments (Figure S4D,E, Supporting Information). These data indicate that CRABP2 enhances mitophagy by downregulating AFG3L2 and that mitophagy is the primary pathway through which CRABP2 suppresses CRLM.

### 2.5 Cytoplasmic CRABP2 Suppresses CRLM by Interacting with the m‐AAA Domain of AFG3L2 to Enhance PINK1‐Mediated Mitophagy

**Figure 5:**
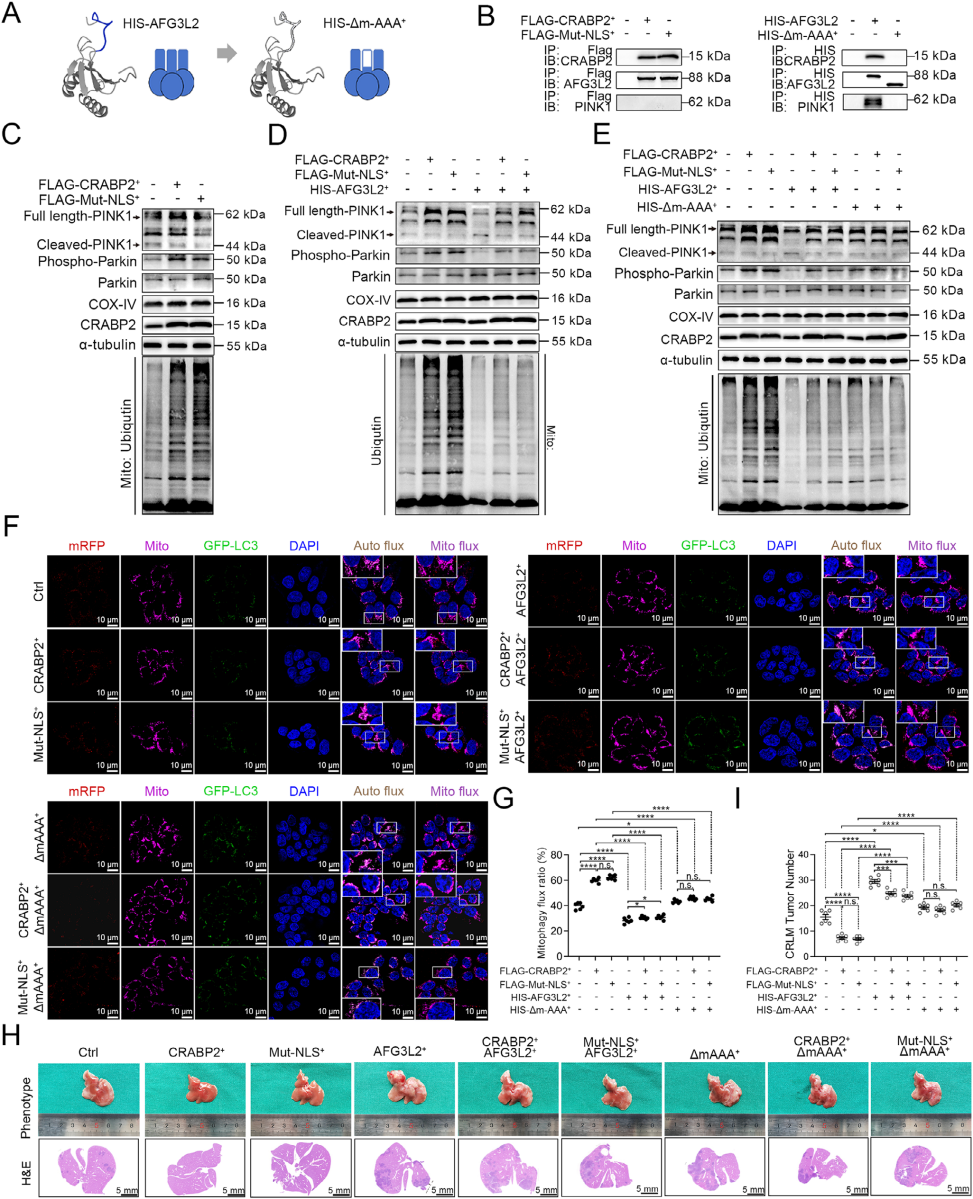
Cytoplasmic CRABP2 suppresses CRLM by interacting with the m‐AAA domain of AFG3L2 to enhance PINK1‐mediated mitophagy. A) Schematic showing the constructs directing cotranslational expression of the His‐AFG3L2 and His‐AFG3L2(Δm‐AAA) proteins. B) Interactions between CRABP2, AFG3L2, and PINK1 in DLD‐1 cells overexpressing CRABP2 or CRABP2‐Mut‐NLS and His‐AFG3L2 or His‐AFG3L2(Δm‐AAA). C–E) Immunoblot analyses measuring the expression of full‐length PINK1, cleaved PINK1, Phospho‐Parkin (Ser65), Parkin, mitochondrial ubiquitin, COX‐IV, and α‐tubulin in DLD‐1 cells overexpressing CRABP2 or CRABP2‐Mut‐NLS and His‐AFG3L2 or His‐AFG3L2(Δm‐AAA). F,G) Mitophagic flux in DLD‐1 cells overexpressing CRABP2 or CRABP2‐Mut‐NLS with/without His‐AFG3L2 or His‐AFG3L2(Δm‐AAA). Levels of mitophagic flux are quantified in (G). Yellow fluorescence indicates LC3 association with the autophagosome, and red fluorescence indicates LC3 association with the autolysosome. H,I) Phenotype and H&E staining of liver tissue from mice injected with *CRABP2* ^+^, *CRABP2‐Mut‐NLS* ^+^, or control (*Ctrl*) cells with or without overexpression of His‐AFG3L2 or His‐AFG3L2(Δm‐AAA). Original magnification ×1 (H), scale bar = 5 mm; ×63 (F), scale bar = 10 µm. The data are presented as the mean ± SD. n.s. = not significant, *p < 0.05, ***p < 0.001, ****p < 0.0001, as analyzed by ANOVA with Tukey's HSD test (G, I).

AFG3L2 is associated with classical PINK1‐mediated mitophagy—a functional activity reported to be mediated by the AFG3L2 m‐AAA region.^[^ ^21^ ^]^ Based on this finding, we constructed a truncated version of AFG3L2 lacking this domain, AFG3L2(Δm‐AAA), and overexpressed this protein to further elucidate the interactions among CRABP2, AFG3L2, and PINK1. We observed interactions between CRABP2 and AFG3L2 and between AFG3L2 and PINK1 but not between CRABP2 and PINK1 (**Figure** **5**A,B). Immunoblot analysis further revealed that CRABP2‐Mut‐NLS and CRABP2 overexpression induced the accumulation of full‐length PINK1, Parkin phosphorylation, and mitochondrial ubiquitination (Figure 5C), whereas AFG3L2 overexpression reversed this effect (Figure 5D). In contrast, overexpression of AFG3L2(Δm‐AAA) did not affect PINK1 or mitochondrial ubiquitination levels but, rather, abolished PINK1 cleavage, Parkin phosphorylation, and mitochondrial ubiquitination induced by CRABP2‐Mut‐NLS and CRABP2 overexpression (Figure 5E). Immunofluorescence also revealed that overexpression of AFG3L2 but not AFG3L2(Δm‐AAA) reversed the increase in mitophagic flux observed in cells overexpressing CRABP2‐Mut‐NLS or CRABP2. Moreover, overexpression of AFG3L2(Δm‐AAA) overexpression appeared to abolish mitophagy induced in response to CRABP2‐Mut‐NLS or CRABP2 overexpression (Figure 5F,G). Thus, these results suggest that CRABP2 promotes mitophagy by interacting with the m‐AAA domain of AFG3L2 and blocking the AFG3L2‐mediated induction of PINK1 degradation.

In the CRLM mouse model, we found that AFG3L2 overexpression reversed the suppression of CRLM observed in response to CRABP2‐Mut‐NLS or CRABP2 overexpression (Figure 5H,I). However, overexpression of AFG3L2(Δm‐AAA) did not have this same effect. Moreover, consistent with our above findings, overexpression of AFG3L2(Δm‐AAA) abolished the suppression of CRLM induced by CRABP2‐Mut‐NLS or CRABP2 overexpression (Figure 5H,I). Similar results were obtained from in vitro experiments (Figure S4F,G, Supporting Information). Collectively, these findings indicate that the Δm‐AAA region is the critical domain through which AFG3L2 regulates the ability of CRABP2 to maintain PINK1‐mediated mitophagy and inhibit CRLM.

### 2.6 CRABP2 Regulates CRC Progression in the Cytoplasm Via the AFG3L2–SLC25A39 Axis

**Figure 6:**
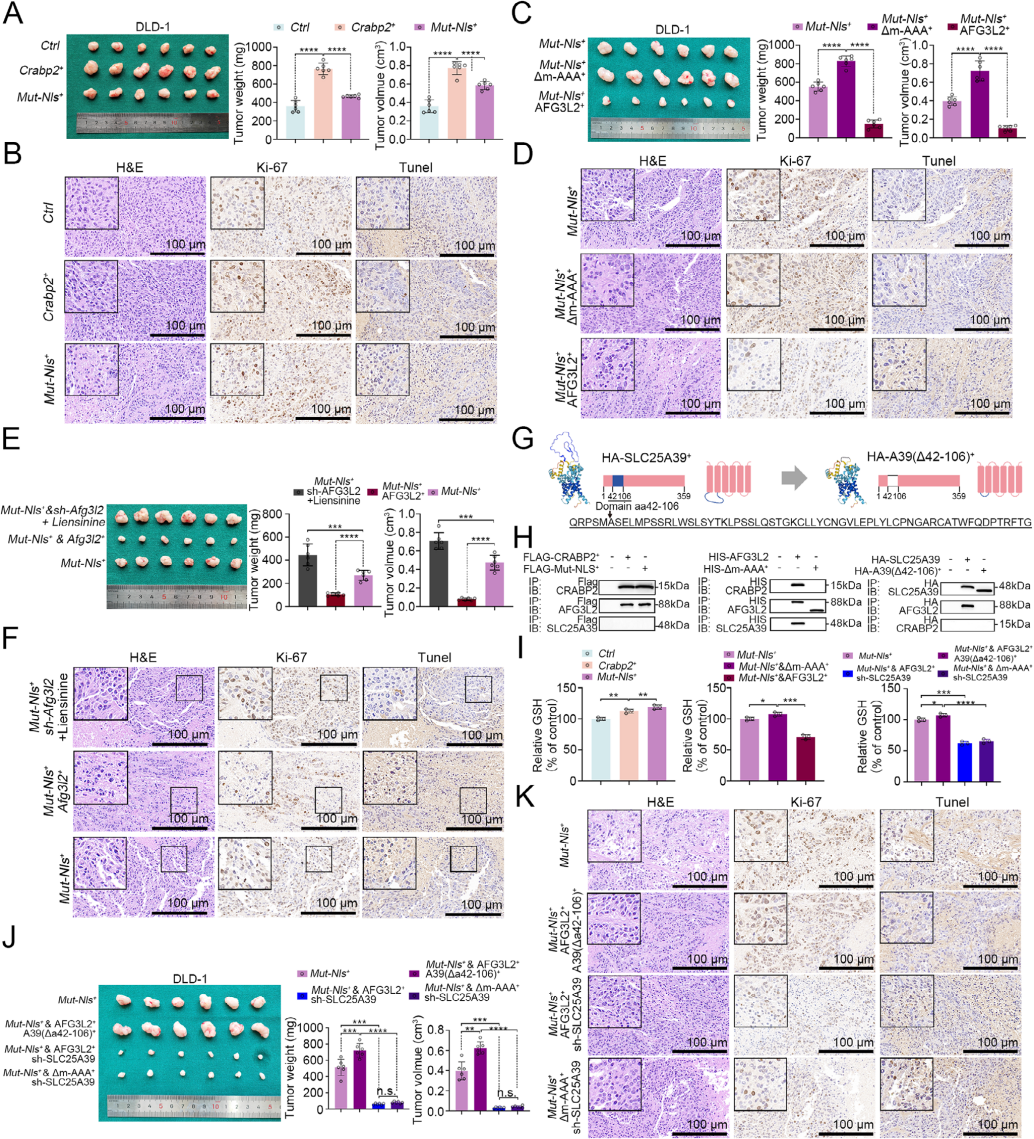
CRABP2 mediates CRC progression in the cytoplasm via the AFG3L2–SLC25A39 axis. A,B) Subcutaneous tumorigenesis assays in nude mice injected with *CRABP2^+^*, *CRABP2‐Mut‐NLS^+^*, or *Ctrl* cells. Tumor weight and volume, H&E staining, immunohistochemical staining for Ki‐67, and TUNEL staining are shown (*n* = 6). C,D) Subcutaneous tumorigenesis assays in nude mice injected with *CRABP2‐Mut‐NLS^+^, CRABP2*‐*Mut‐NLS^+^/AFG3L2(Δm‐AAA)^+^*, or *CRABP2‐Mut‐NLS^+^/AFG3L2^+^* cells. Tumor weight and volume, H&E staining, immunohistochemical staining for Ki‐67, and TUNEL staining are shown (*n* = 6). E,F) Subcutaneous tumorigenesis assays in nude mice injected with *CRABP2‐Mut‐NLS^+^*, *CRABP2‐Mut‐NLS^+^/AFG3L2^+^*, or *CRABP2‐Mut‐NLS^+^/sh‐AFG3L2^+^* cells with or without liensinine feeding. Tumor weight and volume, H&E staining, immunohistochemical staining for Ki‐67, and TUNEL staining are shown (*n* = 6). G) Schematic showing the constructs directing cotranslational expression of HA‐SLC25A39 and HA‐SLC25A39(Δ42–106). H) Co‐IP experiments measuring two‐way interactions between CRABP2, AFG3L2, and SLC25A39 in DLD‐1 cells overexpressing Flag‐CRABP2 or Flag‐CRABP2‐Mut‐NLS, His‐AFG3L2 or His‐AFG3L2(Δm‐AAA), and HA‐SLC25A39 or HA‐SLC25A39(Δ42–106). I) Relative GSH levels in DLD‐1 cells overexpressing CRABP2 or CRABP2‐Mut‐NLS, His‐AFG3L2 or His‐AFG3L2(Δm‐AAA), and HA‐SLC25A39 or HA‐SLC25A39(Δ42–106). J,K) Subcutaneous tumorigenesis assays in nude mice injected with *CRABP2‐Mut‐NLS^+^*, *CRABP2‐Mut‐NLS^+^/AFG3L2^+^/SLC25A39(Δ42–106)^+^, CRABP2‐Mut‐NLS^+^/AFG3L2^+^/sh‐SLC25A39*, or *CRABP2‐Mut‐NLS^+^/AFG3L2(Δm‐AAA)^+^/sh‐SLC25A39* cells. Tumor weight and volume, H&E staining, immunohistochemical staining for Ki‐67, and TUNEL staining are shown (*n* = 6). Original magnification ×40 (B, D, F, K), scale bar = 10 µm. The data are presented as the mean ± SD. n.s. = not significant, *p < 0.05, **p < 0.01, ***p < 0.001, ****p < 0.0001, as analyzed by ANOVA with Tukey's HSD test (A, C, E, I, J).

We next tested the constructs described above in our subcutaneous tumorigenesis model, revealing that CRABP2‐Mut‐NLS overexpression had tumor‐promoting effects (**Figure** **6**A,B; Figure S5A, Supporting Information). Moreover, whereas AFG3L2 overexpression blocked these effects, overexpression of AFG3L2(Δm‐AAA) markedly aggravated tumor growth (Figure 6C,D; Figure S5B, Supporting Information). Given that mitophagy can limit proliferation and promote apoptosis,^[^ ^5^, ^6^ ^]^ we treated mice with the mitophagy inhibitor liensinine to test the role of mitophagy in tumor progression in vivo. We found that liensinine aggravated tumor growth when administered in combination with CRABP2‐Mut‐NLS overexpression (Figure 6E,F; Figure S5C, Supporting Information), with consistent results observed in vitro (Figure S5D–G, Supporting Information). Intriguingly, these findings suggest the existence of another mechanism affecting cell proliferation within the cytoplasm distinct from the nuclear RB1 pathway.

Of note, it was previously reported that AFG3L2 influences cell proliferation through interaction with SLC25A39, a protein that regulates and maintains the stability of the antioxidant glutathione (GSH) in mitochondria.^[^ ^22^ ^]^ GSH functions as a free radical scavenger and detoxifier in cells and is closely related to tumor progression, playing crucial roles in cell proliferation, oxidative stress response, and the maintenance of protein function.^[^ ^23^ ^]^ Studies further identified the functional domain of SLC25A39 in the region spanning residues 42 and 106.^[^ ^22^, ^24^ ^]^ Based on these observations, we generated a full‐length and modified SLC25A39 overexpression construct, the latter containing a deletion of the predicted functional region (Δ42–106) (Figure 6G). In co‐IP experiments, we observed direct interactions between CRABP2 and AFG3L2, as well as between AFG3L2 and SLC25A39, but not between CRABP2 and SLC25A39. Moreover, the Δ42–106 sequence in SLC25A39 was required for interaction with AFG3L2, whereas the Δm‐AAA region in AFG3L2 was required for interaction with SLC25A39 (Figure 6H). In addition, we found that both *CRABP2^+^* and *CRABP2‐Mut‐NLS* ^+^ cells contained higher GSH levels than control cells, whereas overexpression of AFG3L2, SLC25A39 knockdown, or treatment with the GSH inhibitor HY‐106376A^[^ ^25^ ^]^ reversed this effect (Figure 6I,J; Figure S6A, Supporting Information).

We then performed additional subcutaneous tumorigenesis experiments to further explore the proliferative mechanisms acting within the cytoplasm. Results showed blocking the CRABP2–AFG3L2–SLC25A39 interaction led to reduced tumor weight and size (Figure 6J,K; Figure S6B, Supporting Information), with consistent results obtained from in vitro experiments (Figure S6C,D, Supporting Information). Together, these findings identify the AFG3L2–SLC25A39 axis as a mechanism by which cytoplasmic CRABP2 promotes tumor progression in CRC cells.

### 2.7 CRABP2 is Upregulated in CRC and Exhibits Localization Patterns With Distinct Prognostic Implications for CRC Progression and Metastasis

**Figure 7:**
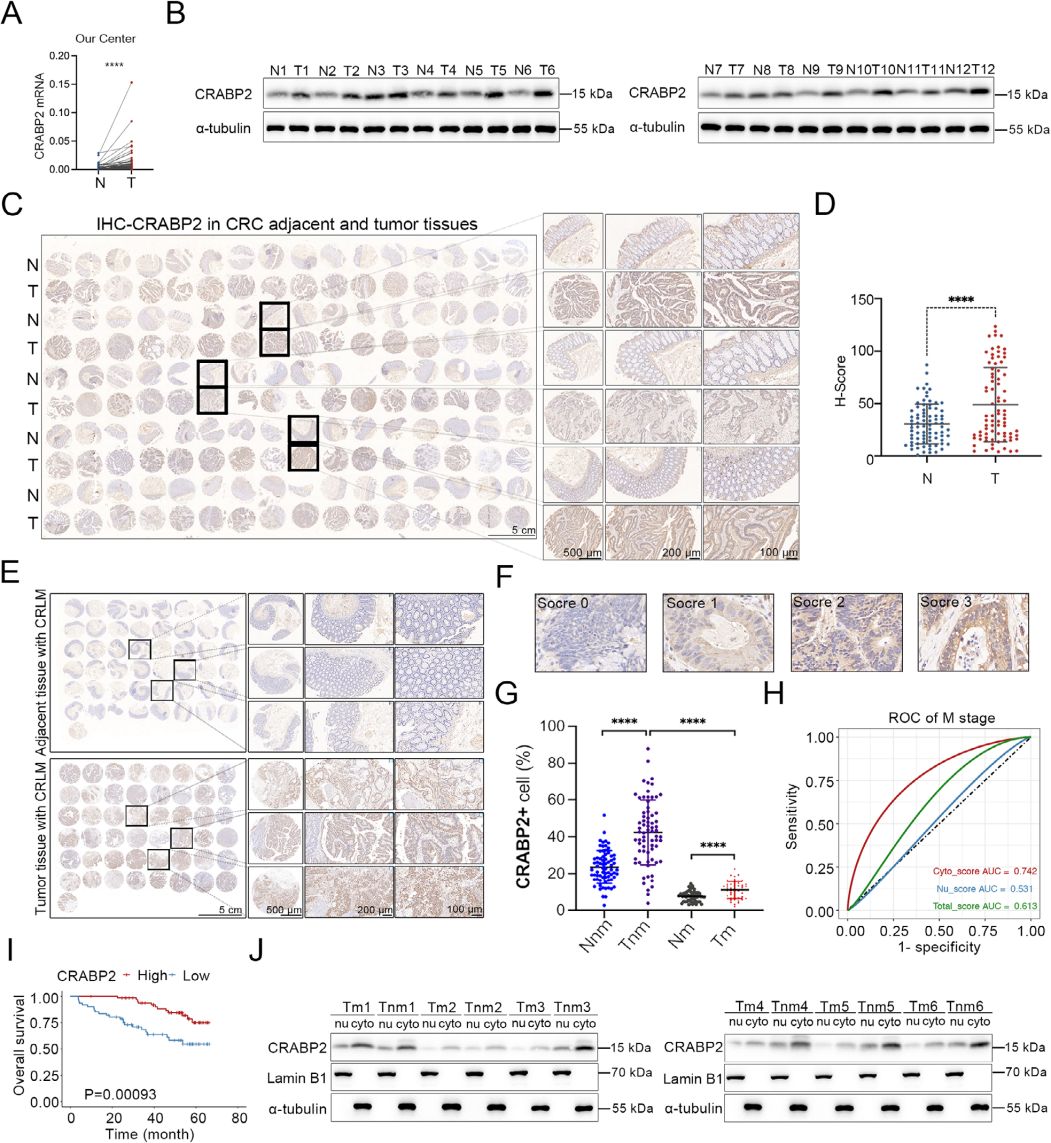
CRABP2 is significantly upregulated in CRC and exhibits localization patterns with distinct prognostic implications for the progression and metastasis of CRC. A) *CRABP2* mRNA levels measured by qRT‐PCR in tumor tissues from CRC patients with/without metastasis. B) Immunoblot analysis measuring CRABP2 and α‐tubulin levels in tumor tissues from CRC patients in (A). C) Immunohistochemical staining for CRABP2 in 80 tissue microarray samples from patients without CRLM. D) H‐Scores for CRABP2 protein levels in the 80 tissue microarray samples in (C). E) Immunohistochemical staining for CRABP2 in 48 tissue microarray samples from patients with CRLM. F) Scores (ranging from 0–4) for CRABP2 protein levels in 128 tissue microarray samples. G) Immunohistochemical staining for CRABP2 in 128 tissue microarray samples. H) ROC curves for cytoplasmic, nuclear, and total CRABP2 scores predicting M‐stage in 128 tissue microarray samples. I) Kaplan–Meier survival curve for the patients described in Tables S3–S5 (Supporting Information) showing a significant difference in OS in those with high versus low CRABP2 expression. J) Immunoblot analysis measuring expression of cytoplasmic and nuclear CRABP2 and α‐tubulin in tumor tissues from the CRC patients with and without metastasis. Original magnification ×0.8 (C, E), scale bar = 5 cm, ×1 (C, E), scale bar = 500 µm, ×5 (C, E), scale bar = 200 µm, ×20 (C, E), scale bar = 100 µm. The data are presented as the mean ± SD. n.s. = not significant, ****p < 0.0001, as analyzed by *t*‐test (A, D) and by ANOVA with Tukey's HSD test (G). The *p*‐values for K–M survival curves were calculated by the log–rank test.

To better understand the role of CRABP2 in CRC in vivo, we next analyzed paired samples from CRC patients (Tables S3–S5, Supporting Information) and observed a significant upregulation of *CRABP2* mRNA in CRC samples relative to non‐tumor tissue (**Figure** **7**A). Immunoblotting and immunohistochemistry analysis further showed that CRABP2 protein levels are increased in CRC samples versus normal tissue (Figure 7B–E).

Notably, Kaplan–Meier survival curve analysis revealed that increased CRABP2 expression was significantly predictive of poorer overall survival (OS) in non‐metastatic TCGA‐colon adenocarcinoma/rectal adenocarcinoma (COADREAD) patients (Figure S6A, Supporting Information, *p* = 0.048). In contrast, no significant association between CRABP2 expression and OS was observed for the overall TCGA‐COADREAD cohort (Figure S6B, Supporting Information, *p* = 0.13). These findings suggest that CRABP2 may primarily promote the progression of tumors that have not yet metastasized, leading to worse outcomes.

We then performed immunohistochemistry evaluating CRABP2 protein expression in 128 tissue microarray samples to assess correlation with the Tumor‐Node‐Metastasis (TNM) stage. Results of receiver operating characteristic curve (ROC) analysis revealed that cytoplasmic CRABP2 (Cyto‐CRABP2) had the highest predictive value for the M‐stage, with an AUC of 0.742, followed by nuclear CRABP2 (Nu‐CRABP2) and total CRABP2, with AUCs of 0.531 and 0.613, respectively (Figure 7E–I; Figure S6C, Supporting Information). These observations suggest that levels of Cyto‐CRABP2 are more strongly predictive for tumor metastasis than those of either Nu‐CRABP2 or total CRABP2.

Correlation analysis further revealed Cyto‐CRABP2 expression to be negatively correlated with the M‐stage (*R* = −0.438, *p* < 0.001), suggesting that higher cytoplasmic expression is associated with less advanced metastatic disease. In contrast, Nu‐CRABP2 expression was positively correlated with the T‐stage (*R* = 0.232, *p* = 0.008), indicating that higher levels of nuclear expression are linked to more advanced primary tumors. Total CRABP2 expression also showed a significant negative correlation with M‐stage (*R* = −0.203, *p* = 0.021; Figure S6D, Supporting Information). Lastly, we performed immunoblot analysis confirming CRABP2 expression in the nucleus and cytoplasm of tumor cells from patients with and without CRLM, respectively (Figure 7J). In total, these findings suggest that cytoplasmic versus nuclear subcellular localization of CRABP2 may have distinct prognostic and functional roles in the progression and metastasis of CRC.

## 3 Discussion

CRC ranks among the leading causes of cancer incidence and mortality worldwide.^[^ ^1^ ^]^ Patients with this disease exhibit poor prognosis and high mortality rates, which are primarily attributed to local progression and distant metastasis, with the liver being the most common metastatic site due to its unique blood supply. Approximately half of CRC patients develop CRLM; however, the molecular mechanisms underlying this phenomenon are not fully understood.^[^ ^2^ ^]^ Here, we identified a dual role for CRABP2—a cytoplasmic‐to‐nuclear transport protein linked with various cancers—in CRC progression and CRLM through its multifaceted and complex interactions in the cytoplasm and nucleus.

Tumorigenesis is a multistep process involving genetic mutations and alterations in numerous molecular mechanisms that influence tumor proliferation and apoptosis, thereby impacting tumor progression.^[^ ^26^ ^]^ Here, we confirmed that CRABP2 is upregulated in tumor tissue from CRC patients, with higher levels of cytoplasmic relative to nuclear CRABP2, and that cytoplasmic and nuclear localization of CRABP2 lead to different biological effects. Specifically, our data show that nuclear CRABP2 interacts with and downregulates RB1, leading to enhanced CRC cell proliferation, suppression of apoptosis, and aggravated CRC progression in vivo and in vitro (Figures 1 and 2; Figures S1 and S2, Supporting Information). In addition, by binding to the m‐AAA domain of AFG3L2, cytoplasmic CRABP2 can block the degradation of SLC25A39, leading to increased GSH levels, which, in turn, promote CRC cell proliferation and inhibition of apoptosis (Figure 6; Figure S5, Supporting Information). Thus, our findings indicate that nuclear and cytoplasmic CRABP2 aggravates tumorigenesis via the CRABP2–RB1 axis and the CRABP2–AFG3L2–SLC25A39–GSH axis, respectively. These findings are consistent with prior studies reporting a role for CRABP2 in promoting proliferation and inhibiting apoptosis in various types of tumors.^[^ ^12^, ^27^ ^]^

Mitophagy is a critical cellular pathway regulated by Parkin and PINK1 that is involved in numerous processes, including cancer, immunity, and tissue loss. In colon tumors, PINK1 was shown to inhibit tumor growth by activating p53 to alter metabolic reprogramming.^[^ ^5^, ^6^ ^]^ Interestingly, we also uncovered a paradoxical effect of cytoplasmic CRABP2 on mitophagy and CRLM, distinct from its nuclear role in promoting CRC cell proliferation. Specifically, CRABP2 suppressed CRLM by interacting with the m‐AAA domain of AFG3L2, which blocks PINK1 degradation to maintain mitophagy (Figures 3, 4, 5; Figures S3 and S4, Supporting Information). Of note, a similar dual role for CRABP2 in breast cancer metastasis under different conditions has also been reported,^[^ ^12c^ ^]^ with several studies identifying opposing effects on proliferation and metastasis in various cancers.^[^ ^28^ ^]^ Overall, however, our data suggest that the pro‐tumorigenesis function of CRABP2 is greater than the anti‐tumorigenesis effects induced by PINK1‐mediated mitophagy (Figure 6; Figures S5 and S6, Supporting Information). Thus, the overall effect of CRABP2 in CRC tumorigenesis appears to be predominantly pro‐proliferative and anti‐apoptosis.

Tumor initiation, progression, and metastasis involve complex interactions among numerous molecules within dynamic networks.^[^ ^29^ ^]^ These interactions can exert divergent effects under different biological conditions, with underlying molecular networks that are in constant flux. Thus, any shifts in the balance among the many cancer‐related pathways can drive distinct pathological outcomes.^[^ ^30^ ^]^ For example, the Notch signaling pathway functions as a molecular switch in cancer, acting as either an oncogene or tumor suppressor depending on the context.^[^ ^31^ ^]^ Moreover, similar to the dual, location‐dependent function of CRABP2, CDK5 acts as a tumor suppressor when localized in the nucleus but functions as an oncogene when present in the cytoplasm.^[^ ^31^ ^]^

From a clinical perspective, we found that CRABP2 is significantly upregulated in CRC tissues (Figure 7A–E; Figure S1, Supporting Information). Kaplan–Meier survival analyses further indicate that CRABP2 primarily promotes tumor progression in non‐metastatic stages, leading to worse prognoses (Figure S7, Supporting Information). In addition, immunohistochemical analysis of tissue microarrays from patients at our center revealed that Cyto‐CRABP2 has strong predictive value for tumor metastasis, with Nu‐CRABP2 and total‐CRABP2 serving as relatively weaker predictors. Our correlation analyses also show that higher cytoplasmic expression is associated with lower metastatic potential, whereas higher nuclear expression correlates with more advanced primary tumors. Moreover, total CRABP2 expression was significantly correlated with the M‐stage (Figure S7, Supporting Information). Thus, our findings suggest that the subcellular localization of CRABP2 has distinct prognostic implications and functional roles in the progression and metastasis of CRC.

In conclusion, our study reveals a dual localization‐dependent role for CRABP2 in CRC progression and metastasis, highlighting its complex molecular interactions and cellular functions. In the nucleus, CRABP2 promotes CRC progression by interacting with and inhibiting RB1, whereas in the cytoplasm, CRABP2 suppresses CRLM by interacting with the m‐AAA domain of AFG3L2, thereby blocking PINK1 degradation and maintaining mitophagy. However, cytoplasmic CRABP2 also promotes CRC progression by preventing AFG3L2‐mediated degradation of SLC25A39. Consistent with these distinct functions, we further show that the subcellular localization of CRABP2 has differential effects on the progression and liver metastasis of CRC. These findings shed light on the complex mechanisms underlying CRC pathogenesis and suggest the potential for CRABP2 to be leveraged as both a prognostic indicator and a candidate for targeted therapy.

## 4 Experimental Section

### Clinical Specimens

Paired primary CRC and liver metastasis tissues were obtained from the First Affiliated Hospital of Nanjing Medical University (Nanjing, China). Liver metastasis tissues were collected from CRC patients undergoing simultaneous surgery, none of whom had received prior neoadjuvant chemoradiotherapy. Fresh tissue samples were obtained for quantitative reverse transcription (qRT)‐PCR and immunoblot analysis. In addition, tissue microarrays containing 80 and 48 pairs of primary tumors were constructed by Servicebio (Wuhan, China). Pertinent clinical information for all patients was detailed in Tables S3–S5, Supporting Information. This study was approved by the Ethics Committee of the First Affiliated Hospital of Nanjing Medical University, and all patients provided written informed consent (2022‐SRFA‐142).

### Conditional Knockout Mice

CRABP2 conditional knockout mice (*Crabp2* ^ΔIEC^) were generated using Cre recombinase expressed under the villin‐1 (*VIL1*) promoter, which was specific to intestinal epithelial cells. Mice carrying the floxed allele (*Crabp2* ^flox/flox^) were crossed with *VIL1*‐Cre mice to obtain *Crabp2* ^ΔIEC^ progeny. All mice were on a C57BL/6 background.

### Cell Culture

Human cell lines, including DLD‐1, SW480, SW620, RKO, HCT 116, NCM460, and HEK‐293T cells, were purchased from the Cell Bank of Type Culture Collection of the Chinese Academy of Sciences (Shanghai, China). All cell lines were routinely cultured in the recommended media supplemented with 10% fetal bovine serum and 1% penicillin/streptomycin at 37 °C in a humidified atmosphere containing 5% CO_2_.

### Cell Transfection

Lentiviral constructs expressing shRNAs targeting *CRABP2*, *AFG3L2*, or *SLC25A39* were synthesized by Genomeditech (Shanghai, China). The full‐length *CRABP2*, *AFG3L2*, and *RB1* sequences were synthesized using genome‐editing technology and subcloned into lentiviral and adenoviral vectors by Genomeditech. A series of *CRABP2*, *AFG3L2*, and *SLC25A39* mutant plasmids were synthesized by Genomeditech. Plasmid transfection was performed using Lipofectamine 3000 (Invitrogen, Thermo Fisher Scientific, Waltham, MA, USA), and transfection efficiency was verified using qRT‐PCR and immunoblotting. The sequences of all shRNAs were provided in Supplementary Tables.

### Ectopic Expression and Gene Silencing of CRABP2

Constructs for overexpression were subcloned into the lentiviral vector PGMLV‐CMV‐MCS‐PGK‐Puro. Similarly, shRNA sequences specifically targeting CRABP2, AFG3L2, or SLC25A39 were subcloned into the lentiviral vector pLKO.1‐puro. The *CRABP2‐Mut‐NLS^+^* construct directs the overexpression of Flag‐tagged CRABP2 containing mutations at K21, R30, and K31 to K21A/R30A/K31A and was provided by Genomeditech. The *CRABP2‐Mut‐NES^+^* construct directs the overexpression of Flag‐tagged CRABP2 with mutations in the domain spanning V22 to L27 and was provided by Genomeditech. The *His‐AFG3L2(Δm‐AAA)^+^* construct directs the overexpression of His‐tagged AFG3L2 with an m‐AAA domain deletion and was provided by Genomeditech. The *His‐SLC25A39(*Δ*42–106)^+^* construct directs the overexpression of His‐tagged SLC25A39 with an m‐AAA domain deletion and was provided by Genomeditech.

Overexpression and knockdown efficiencies are shown in Figure S1I, Supporting Information. The CRABP2‐specific shRNA was selected exhibiting the highest knockdown efficiency for further experiments. Recombinant lentiviruses were produced by co‐transfecting 293T cells with the lentiviral expression plasmid and packaging plasmids using Lipofectamine 3000 transfection reagent (Invitrogen; #L3000015) according to the manufacturer's protocol. Lentiviruses were purified by ultracentrifugation and filtered for use. Lentiviruses stably expressing *CRABP2^+^*/*CRABP2‐Mut‐NLS^+^*/*His‐AFG3L2^+^*/*His‐AFG3L2(Δm‐AAA)^+^/His‐SLC25A39^+^*/*His‐SLC25A39(Δ42–106)^+^*/*RB1^+^*/*sh‐CRABP2/sh‐RB1*/*sh‐AFG3L2* were transfected to cells cultured in six‐well dishes using polybrene (10 µg mL^−1^) for 24 h.

### Animal Models

For all animal experiments, mice were randomly assigned to groups, each containing *n* = 6 animals. Confounders were controlled by randomizing the order of treatments. For the AOM‐DSS model, male *Crabp2* ^ΔIEC^ and *Crabp2* ^flox/flox^ mice were administered azoxymethane (AOM, 12 mg k^−1^g body weight; Sigma, USA) via intraperitoneal injection at 8 weeks of age. At 1 week after AOM administration, mice were treated with 2% dextran sulfate sodium (DSS, MP Biologicals, USA) in drinking water for one week, followed by 2 weeks of normal drinking water. This cycle was repeated three times. Mice were sacrificed after 16 weeks to observe and record colorectal tumor development. AAV‐9 intervention was performed at 6 weeks of age, with the remaining procedures as described above.

Subcutaneous tumor and liver metastasis models were established in 6‐week‐old male BALB/c nude mice. For the subcutaneous tumor model, 1 × 10^6^ stably transfected DLD‐1 or SW480 cells, along with control cells, were injected into the inguinal region of the mice. After 4 weeks, mice were sacrificed, and the tumors were excised for hematoxylin and eosin (H&E) staining and immunohistochemical analysis. The liver metastasis model was established by injecting 1 × 10^6^ luciferase‐labeled cells into the distal spleen of anesthetized mice. After 4 weeks, bioluminescent images were obtained using the IVIS Spectrum Imaging System (PerkinElmer, USA) following intraperitoneal injection of 150‐mg kg^−1^ D‐luciferin (Goldbio, USA). Mice were sacrificed under anesthesia, and their livers were collected for further analysis. All animal experiments were approved by the Animal Care and Use Committee of Nanjing Medical University and conducted in accordance with NIH guidelines (IACUC‐2309022).

### TEM

Cells and tissue samples were fixed with 3% glutaraldehyde and 1% osmium tetroxide. After further processing, samples were examined under a JEM‐1400FLASH transmission electron microscope (JEOL, Japan).

### Mitochondria Isolation

Collected cells and tissues were ground with phosphate‐buffered saline (PBS), and mitochondria were isolated using the Mitochondria Isolation Kit (Beyotime, China; # C3601).

### Mitophagy Model

DLD‐1/SW480 cells were treated with the oxidative phosphorylation (OXPHOS) uncoupler CCCP (10 µ;m) in vitro for 2 h. CCCP induces PINK1 activation and promotes Parkin phosphorylation at serine 65, both of which lead to mitophagy induction.

### Mitophagy/Autophagy Flux Assay

Mitophagy/autophagy flux was assessed in DLD‐1 and SW480 cells using the Premo Autophagy Tandem Sensor mRFP‐GFP‐LC3 Kit (Thermo Fisher Scientific; #P36239) following the manufacturer's instructions. Briefly, 30 000 cells from tissue or 20 000 CRC cells treated or untreated with CCCP were seeded in 24‐well plates and transfected with mRFP‐GFP‐LC3 for 24 h. Cells were then washed with PBS and fixed in 4% paraformaldehyde for 30 min. After permeabilization and blocking, sections were incubated with Anti‐TOMM20 (Abcam #ab186735) at 4 °C overnight. The sections were then washed with PBS three times and stained with Alexa 647‐conjugated anti‐rabbit secondary antibody for 2 h at room temperature. The nucleus was counterstained with 4′,6‐diamidino‐2‐phenylindole (DAPI). With this construct, yellow fluorescence indicates LC3 association with the autophagosome, whereas red fluorescence indicates LC3 association with the autolysosome. All images were captured using an inverted confocal fluorescent microscope (Leica Microsystems CMS GmbH, TCS SP8) and software (Leica Application Suite X, 3.6.20104.0).

### Immunofluorescence

For immunofluorescence experiments, cells from different treatment groups were seeded onto confocal dishes and fixed with 4% paraformaldehyde for 30 min. After permeabilization and blocking, fixed cells were incubated overnight at 4 °C with primary antibodies (Mouse Polyclonal anti‐AFG3L2, ab68023; Mouse Monoclonal anti‐RB1; Rabbit Polyclonal anti‐CRABP2 10225‐1‐AP; Rabbit monoclonal anti‐TOMM20, ab186735), followed by incubation with secondary antibodies at room temperature for 2 h. Nuclei were stained using DAPI, and the cells were analyzed using a confocal fluorescence microscope.

### Subcellular Protein Fractionation

Subcellular protein fractionation was performed using the PARIS Kit (AM1921; Thermo Fisher Scientific) according to the manufacturer's instructions. In brief, the collected samples were first incubated with cytoplasmic extract reagent; following centrifugation, the supernatant (cytoplasmic extract) was transferred to a new tube and saved for immunoblot analysis. The insoluble fraction was then resuspended in a cold nuclear extraction reagent, vortexed, and centrifuged, and the supernatant (nuclear extract) was collected for subsequent immunoblot analysis. Lamin‐B1 and α‐Tubulin were used as loading controls for nuclear and cytoplasmic proteins, respectively.

### IP Assays

After washing cells with 2 mL PBS, cell lysates were collected with lysis buffer, incubated on ice for 30 min, and centrifugated at 12 000 rpm for 30 min. The supernatant was collected, and protein concentration was determined using the BCA Protein Assay Kit (Beyotime; #P0012). Proteins were either directly analyzed by immunoblotting as input or used for IP analysis with the Pierce Direct Magnetic IP/Co‐IP Kit (Thermo Fisher Scientific; #88804). In brief, proteins were first incubated with antibodies (Polyclonal anti‐CRABP2 10225‐1‐AP; Rabbit Polyclonal anti‐RB1, Proteintech, 10048‐2‐Ig; Rabbit Polyclonal anti‐AFG3L2, Proteintech, 14631‐1‐AP; Rabbit monoclonal Anti‐PINK1, CST, 6946; Rabbit monoclonal anti‐FLAG, CST, 14793; Rabbit monoclonal anti‐His‐Tag, CST, 12698) and magnetic beads overnight. After collection with a magnetic stand (Thermo Fisher Scientific), beads were washed three times with wash buffer. The beads were then resuspended and heated in a loading buffer, and the supernatant was separated by SDS‐PAGE.

### MS Analysis

Tissue samples were ground in RIPA buffer containing phenylmethanesulfonyl fluoride (PMSF; Beyotime; #P0013) using a High‐Speed Tissue Grinder‐KZ‐II (Wuhan Seville Biotechnology Co., Ltd.). Proteins in cell lysates were immunoprecipitated with anti‐His antibody, separated by SDS‐PAGE, and visualized by Silver Staining (Beyotime; P0017S). Protein in‐gel digestion and nano‐high‐performance liquid chromatography (HPLC) MS/MS were performed as described previously. In addition, proteins from cells were analyzed by nano‐HPLC MS/MS (BGI Shenzhen, China). The acquired MS/MS spectra were searched against the National Center for Biotechnology Information non‐redundant protein sequence database.

### Co‐IP Assays

Two‐way physical interactions between CRABP2, RB1, AFG3L2, and SLC25A39 were examined using an IP/Co‐IP kit (#88804, Thermo Fisher Scientific). Cell lysates were incubated overnight at 4 °C with corresponding primary antibodies. The immune complexes were then incubated with A/G magnetic beads for 1.5 h at room temperature, followed by washing with IP buffer twice and RNase‐free water once. The immunoprecipitated proteins were analyzed by immunoblotting or MS (BGI Shenzhen, China). Antibodies used in this assay were listed in the Supplementary Tables.

### Cell Proliferation, Transwell, and Wound Healing Assays

Cell proliferation was assessed using a Cell Counting Kit‐8 (CCK‐8) (Beyotime). Colony formation, Transwell migration, and wound healing assays were performed as previously described.^[^ ^32^ ^]^

### Flow Cytometry Apoptosis Assays

Cells (3 × 10^5^ per well) were seeded in 6‐well plates and cultured for 48 h. Apoptosis was measured using the Annexin V‐FITC/PI Apoptosis Detection Kit (Vazyme, China) according to the manufacturer's instructions, and apoptosis rates were analyzed using FlowJo software (BD Biosciences, USA).

### Intracellular GSH Content Detection

Cells from different groups were seeded into 24‐well plates and cultured overnight. Intracellular GSH levels were measured using the GSH/GSSG Assay Kit (S0053; Beyotime) according to the manufacturer's instructions. Absorbance was measured at 412 nm to calculate GSH content.

### Immunoblot Analyses and Immunohistochemistry

Cellular and tissue proteins were extracted using RIPA lysis buffer (Beyotime) supplemented with PMSF according to the manufacturer's instructions. Protein concentrations were determined using the BCA Protein Assay Kit (Beyotime). Protein lysates were separated by SDS‐PAGE (Beyotime) and transferred to polyvinylidene fluoride membranes (Millipore, USA). The membranes were then blocked with QuickBlock (Beyotime) for 30 min and incubated overnight at 4 °C with primary antibodies. The next day, the membranes were incubated with secondary antibodies at room temperature for 2 h, and immunoreactive bands were visualized using an enhanced chemiluminescence method. Immunohistochemistry was performed as previously described.^[^ ^33^ ^]^ The antibodies used in this study were listed in the Supplementary Tables.

### RNA Extraction and qRT‐PCR

Total RNA was extracted from tissues and cell lines using TRIzol reagent (Invitrogen) according to the manufacturer's protocol. RNA quality and quantity were assessed using a NanoDrop spectrophotometer. Total RNA was then reverse‐transcribed into cDNA using the HiScript RT Mix (Vazyme), and qRT‐PCR was performed using SYBR Green reagent (Vazyme) and specific primers. The resulting data were analyzed using StepOne software v2.3. All primer sequences were listed in the Supplementary Tables.

### Survival Analysis

Survival analyses and visualizations were performed using the survival package (v3.2.13) in R. Tissue samples were divided into high and low gene expression groups based on their median expression levels. Kaplan–Meier curves for OS were generated using the survival fitting function.

### ROC Curve Analysis

ROC curve analysis was performed to evaluate the diagnostic performance of the identified biomarkers. ROC curves were also generated to assess the sensitivity and specificity of the predictive model. The area under the ROC curve (AUC) was calculated as a quantitative measure of model accuracy.

### Statistical Analysis

Statistical analyses were performed using GraphPad Prism v.10.0 (La Jolla, CA, USA) and SPSS v.25.0 (Chicago, IL, USA). Statistical methods included *t*‐tests, Wilcoxon rank–sum tests, analysis of variance (ANOVA), and chi‐square tests. For post hoc comparisons following ANOVA, Tukey's honestly significant difference test was used. For Kaplan–Meier survival curves, *p*‐values were calculated using the log–rank test. All experiments were repeated at least three times, and data were expressed as mean ± standard deviation (SD); *p*‐values <0.05 were considered statistically significant.

## Conflict of Interest

The authors declare no conflict of interest.

## Author Contributions

C.T., S.Y., C.Z., and R.Z. contributed equally to this work. Y.S., K.J., J.T., and C.T. conceived and designed the experiments; C.T., R.Z., X.W., S.Y., and D.Z. performed the experiments and bioinformatics analysis; C.T., C.Z., C.C., X.W., Q.S., H.X., and H.N. collected the human specimens; C.T., S.Y., Y.Z., and D.J. analyzed the data; C.T., Y.S., K.J., S.Y., and J.T. wrote the manuscript. All authors have read, revised, and approved the final manuscript. Y.S., K.J., and J.T. are responsible for the overall content as the guarantors.

## Supporting information



Supporting Information

## Data Availability

The data that support the findings of this study are available from the corresponding author upon reasonable request.
