# Supplementary material for: Dual Role of CRABP2 in Colorectal Cancer: Oncogenesis via Nuclear RB1 and Cytoplasmic AFG3L2/SLC25A39 Axis, While Limiting Liver Metastasis through Cytoplasmic AFG3L2/PINK1/Parkin‐Mediated Mitophagy
Source: Adv Sci (Weinh). 2025 Apr 30;12(23):2500552. doi: 10.1002/advs.202500552 (PMC12199395; doi:10.1002/advs.202500552)
Supplement: Supplementary file 1 — Supporting Information [file ADVS-12-2500552-s001.docx]

**Title:** Dual Role of CRABP2 in Colorectal Cancer: Oncogenesis via Nuclear RB1 and Cytoplasmic AFG3L2/SLC25A39 Axis, While Limiting Liver Metastasis Through Cytoplasmic AFG3L2/PINK1/Parkin-Mediated Mitophagy

**Author:** Chuanxin Tian^1,2,3*^, Sheng Yang^1,2,3*^, Chuan Zhang^1,2,3*^, Renzhong Zhu^4*^, Chen Chen^1,2,3^, Xiaowei Wang^1,2,3^, Dongsheng Zhang^1,2,3^, Qingyang Sun^1,2,3^, Hengjie Xu^1,2,3^, Hongxu Nie^1,2,3^, Yue Zhang^1,2,3^, Dongjian Ji^1,2,3^, Junwei Tang^1,2,3#^, Kangpeng Jin^1,2,3#^, Yueming Sun^1,2,3#^

^1^ Department of General Surgery, Colorectal Institute of Nanjing Medical University, The First Affiliated Hospital of Nanjing Medical University, Nanjing, China. 210029.

^2^ Jiangsu Province Engineering Research Center of Colorectal Cancer Precision Medicine and Translational Medicine, Nanjing, China. 210029.

^3^ Collaborative Innovation Center for Cancer Personalized Medicine, Nanjing Medical University, Nanjing, China. 210029.

^4^ Institute of Translational Medicine, Medical College, Yangzhou University, No.136 Jiangyang Road, Yangzhou, China. 215000.

* These authors contributed equally to this work.

# Correspondence and requests for materials should be addressed to [sunyueming@njmu.edu.cn](mailto:sunyueming@njmu.edu.cn) (Yueming Sun)

**Contents:**

Supplementary figures and legends: Pages 2-15

Supplementary tables and legends: Pages 16-30

**Supplementary Figures and Legends:**

**
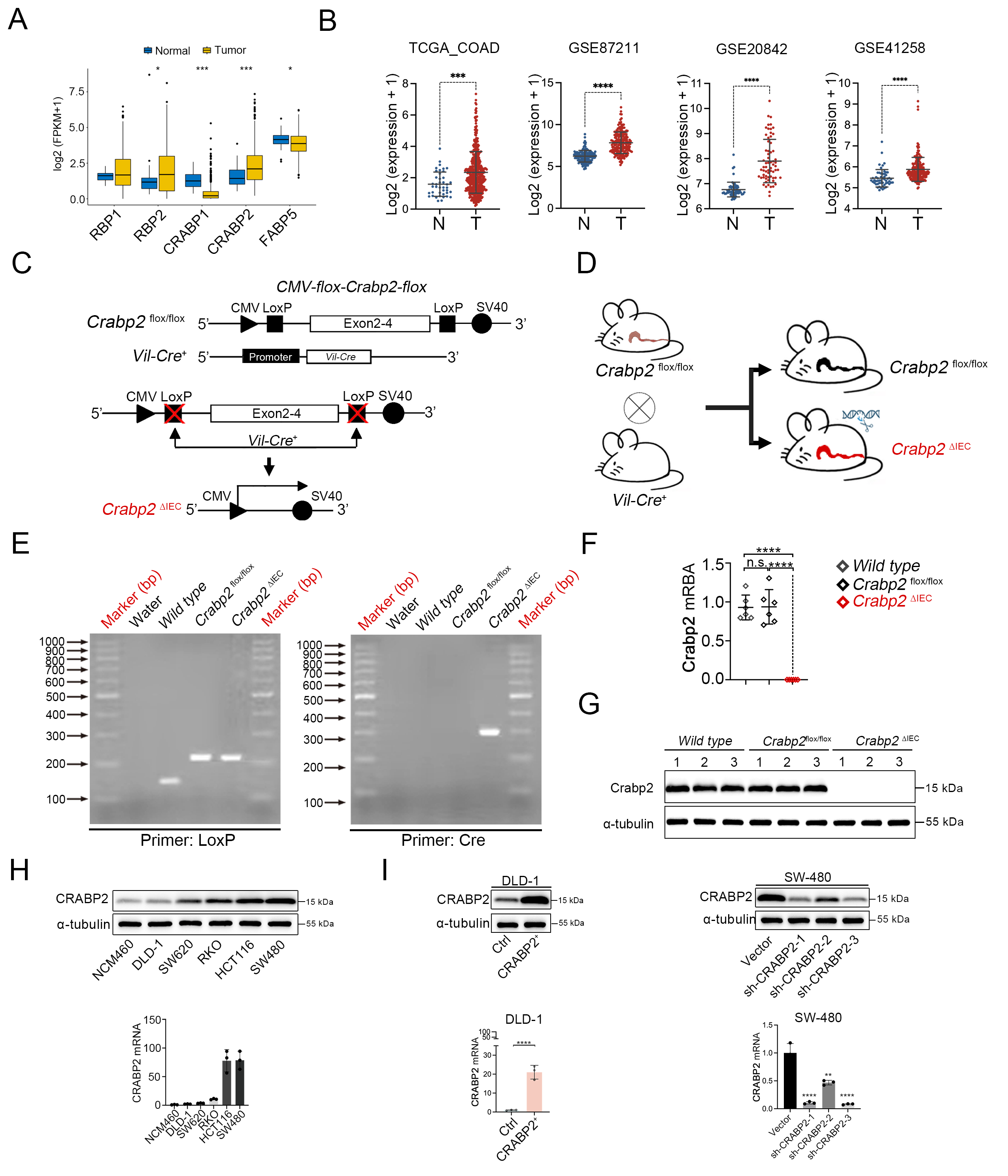
**

**Figure. S1.** CRABP2 was Significantly Upregulated in Colorectal Cancer in TCGA database and GSE database, and the establishment of Colonocyte-specific CRABP2-knockout mice and CRABP2 overexpression and knockdown stable cell. (**A**) the expression of CRBPs family (RBP1, RBP2, CRABP1, CRABP2, FABP5) level in TCGA database. (**B**) the expression of CRABP2 level in TCGA and GEO database. (**C**) CRABP exon 2 to 4 were flanked with loxP sites to generate floxed CRABP2 (*Crabp*^flox/flox^). The related gene type of *Crabp*^flox/flox^ and *Vil-Cre* mice was shown. (**D**) *Crabp*^flox/flox^ mice were crossed with *Vil-Cre* transgenic mice to generate a Colonocyte specific knock out of CRABP2 (hereafter referred to as *Crabp2*^ΔIEC^) respectively. (**E**) The ablation efficiency of CRABP2 in Colonocyte in 6-week-old postnatally mice was confirmed at the DNA level with polymerase chain reaction (PCR). (**F**) Crabp2 mRNA level were detected in primary Colonocyte from wild type, *Crabp*^flox/flox^ and *Crabp2*^ΔIEC^ colon tissues. (**G**) Immunoblotting analysis of Crabp2 in primary Colonocyte from *Wild type*, *Crabp*^flox/flox^ and *Crabp2*^ΔIEC^ colon tissues. (**H**) Immunoblotting analysis and RT-qPCR analysis of CRABP2 in NCM460 and CRC cell lines (DLD-1, SW620, RKO, HCT116 and SW480). (**I**) Immunoblotting analysis and RT-qPCR analysis of CRABP2 in DLD-1 cell line overexpressing CRABP2 and Ctrl, and in SW480 cell line knockdown CRABP2 and vector. The data are presented as the mean ± SD. *p <0.05, ***p <0.001, ****p <0.0001, as analyzed by Wilcoxon rank-sum tests **(A, B)**. n.s. no significant, *p <0.05, **p <0.01, ***p <0.001, ****p <0.0001 as analyzed by t-test **(I)** for comparisons between two groups, and by ANOVA followed by Tukey's honestly significant difference test **(F, H, I)** for comparisons between multiple groups.

**
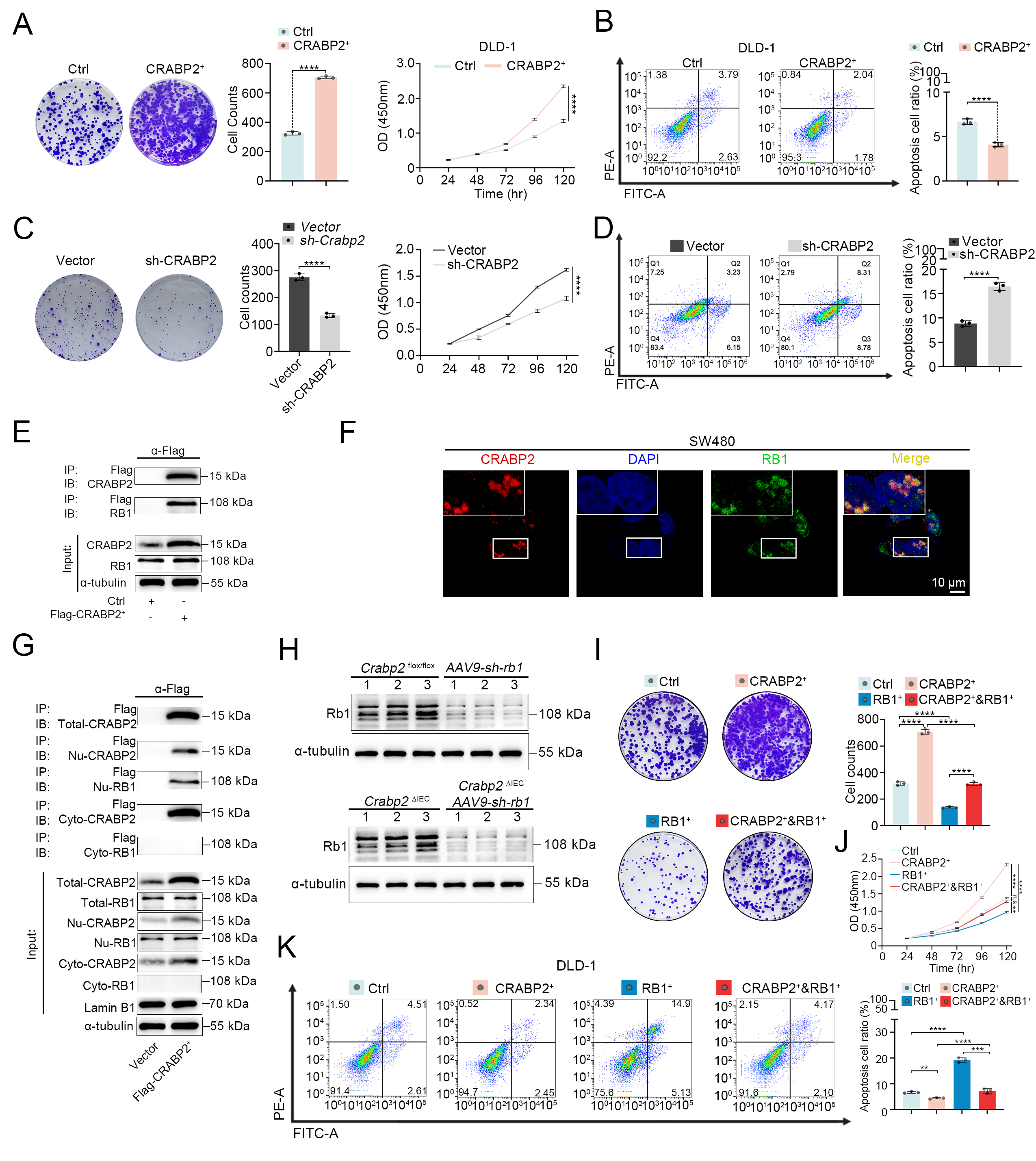
**

**Figure. S2.** CRABP2 promotes CRC Progression in vivo and enhances proliferation, suppresses apoptosis by downregulating RB1 in vitro.
(**A**) Statistical results of plate colony (left) and the CCK-8 assay (right) in Ctrl and CRABP2^+^ groups. (**B**) The flow cytometry analysis of apoptosis in CRABP2^+^ and Ctrl group. **(C**) Statistical results of plate colony (left) and the CCK-8 assay (right) in CRC cells with CRABP2 knockdown and Ctrl. (**D**) The flow cytometry (FCM) analysis of apoptosis in CRABP2 knockdown of SW480 cells. (**E**) Combination of CRABP2 and RB1 by co-immunoprecipitation in DLD-1 cell overexpressing Flag-CRABP2. (**F**) The co-localization of CRABP2 (red) and RB1 (green) detect by immunoprecipitation assays in SW480 cell line. (**G**) Combination of nucleus CRABP2 and nucleus RB1, cytoplasmic CRABP2 and cytoplasmic RB1, and total CRABP2 and total RB1 by co-immunoprecipitation in DLD-1 cell overexpressing Flag-CRABP2. (**H**) Immunoblotting analysis of CRABP2 in *Crabp2*^ΔIEC^ , *Crabp2*^flox/flox^ , *Crabp2*^ΔIEC^ + AAV9-sh-rb1 and *Crabp2*^flox/flox^ + AAV9-sh-rb1 groups (n = 6). (**I**) Statistical results of plate colony in CRABP2 overexpression cells, RB1 overexpression cells and double overexpression cells. (**J**) CCK-8 assay was performed to determine the proliferation rate of CRABP2 overexpression cells, RB1 overexpression cells and double overexpression cells. (**K**) The flow cytometry (FCM) analysis of apoptosis in CRABP2 overexpression cells, RB1 overexpression cells and double overexpression cells. Bar=10 μm. Original magnification ×63 (**F**), Bar=10 μm. The data are presented as the mean ± SD. n.s. no significant, **p <0.01, ****p <0.0001, as analyzed by t-test (**A, B, C, D**)**.** n.s. no significant, **p <0.01, ***p <0.001, ****p <0.0001, as analyzed by ANOVA and Tukey’s honestly significant difference (**I, J, K**).

**
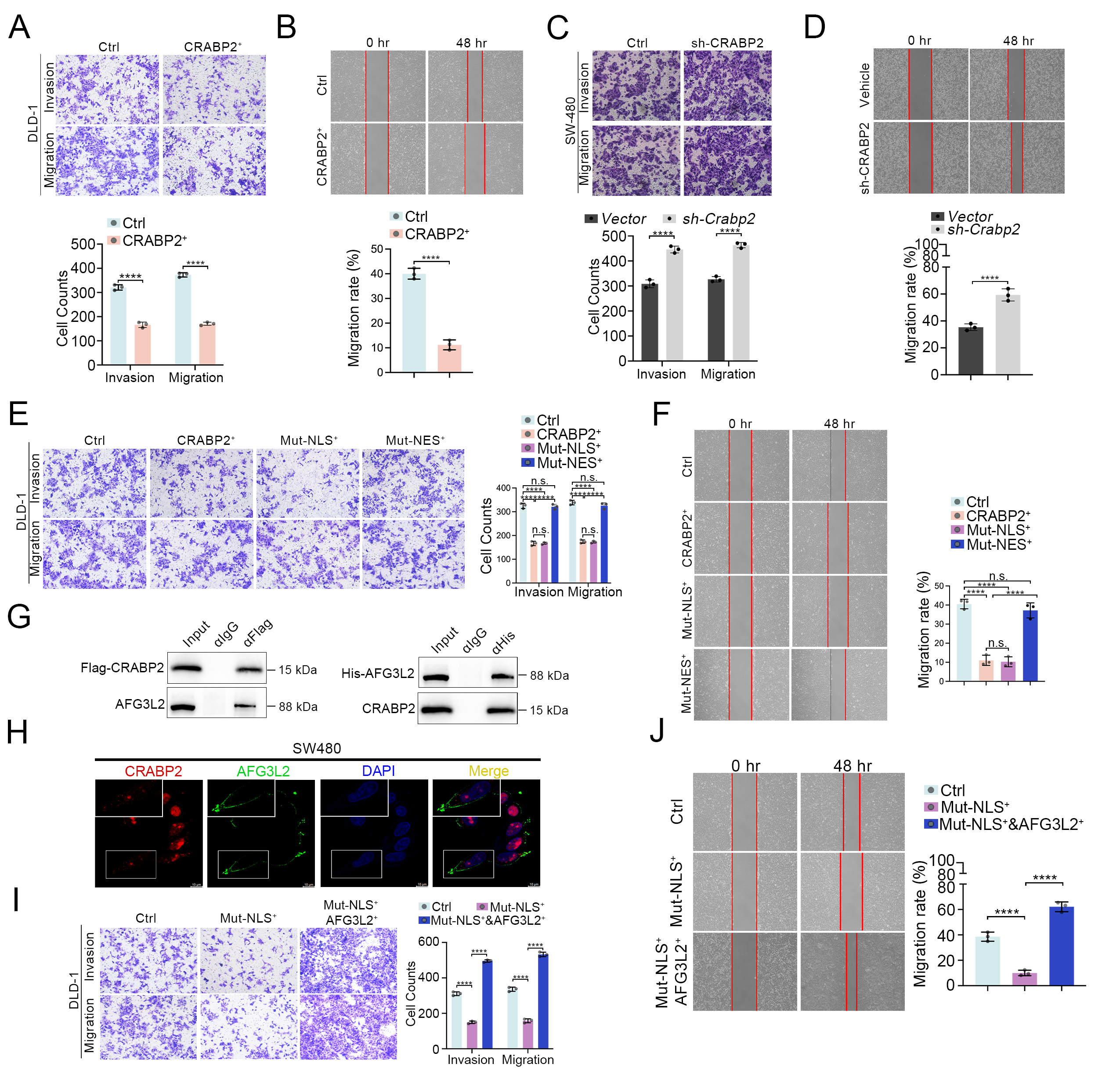
**

**Figure. S3.** Cytoplasmic CRABP2 Suppresses CRLM via Interacting with AFG3L2 to Maintain Mitophagy independently of its nucleus function. (**A**) Transwell assays were used to detect the migration and invasion of CRC cells overexpressing CRABP2 and Ctrl. (**B**) Wound healing assays were used to detect the migration of CRC cells overexpressing CRABP2 and Ctrl. (**C**)Transwell assays were used to detect the migration and invasion of CRC cells with knockdown CRABP2 and Vector. (**D**) Wound healing assays were used to detect the migration of CRC cells with knockdown CRABP2 and Vector. (**E**) Transwell assays were used to detect the migration and invasion of CRC cells overexpressing CRABP2, Mut-NLS, Mut-NES and Ctrl. (**F**) Wound healing assays were used to detect the migration of CRC cells overexpressing CRABP2, Mut-NLS, Mut-NES and Ctrl. (**G**) Interactions of CRABP2, AFG3L2 in DLD-1 cells overexpressing Flag-CRABP2 and HIS-AFG3L2. (**H**)The co-localization of CRABP2 (red) and AFG3L2 (green) detect by immunoprecipitation assays in SW480 cell lines. (**I**) Transwell assays were used to detect the migration and invasion of CRC cells overexpressing Mut-NLS and Mut-NLS&AFG3L2. (**J**) Wound healing assays were used to detect the migration of CRC cells overexpressing Mut-NLS and Mut-NLS&AFG3L2.

Original magnification ×63 (**H**), Bar=10 μm. The data are presented as the mean ± SD. n.s. no significant, ****p <0.0001, as analyzed by t-test (**A, B, C, D**) for comparisons between two groups, and by ANOVA followed by Tukey's honestly significant difference test (**E, F, I, J**) for comparisons between multiple groups.

**
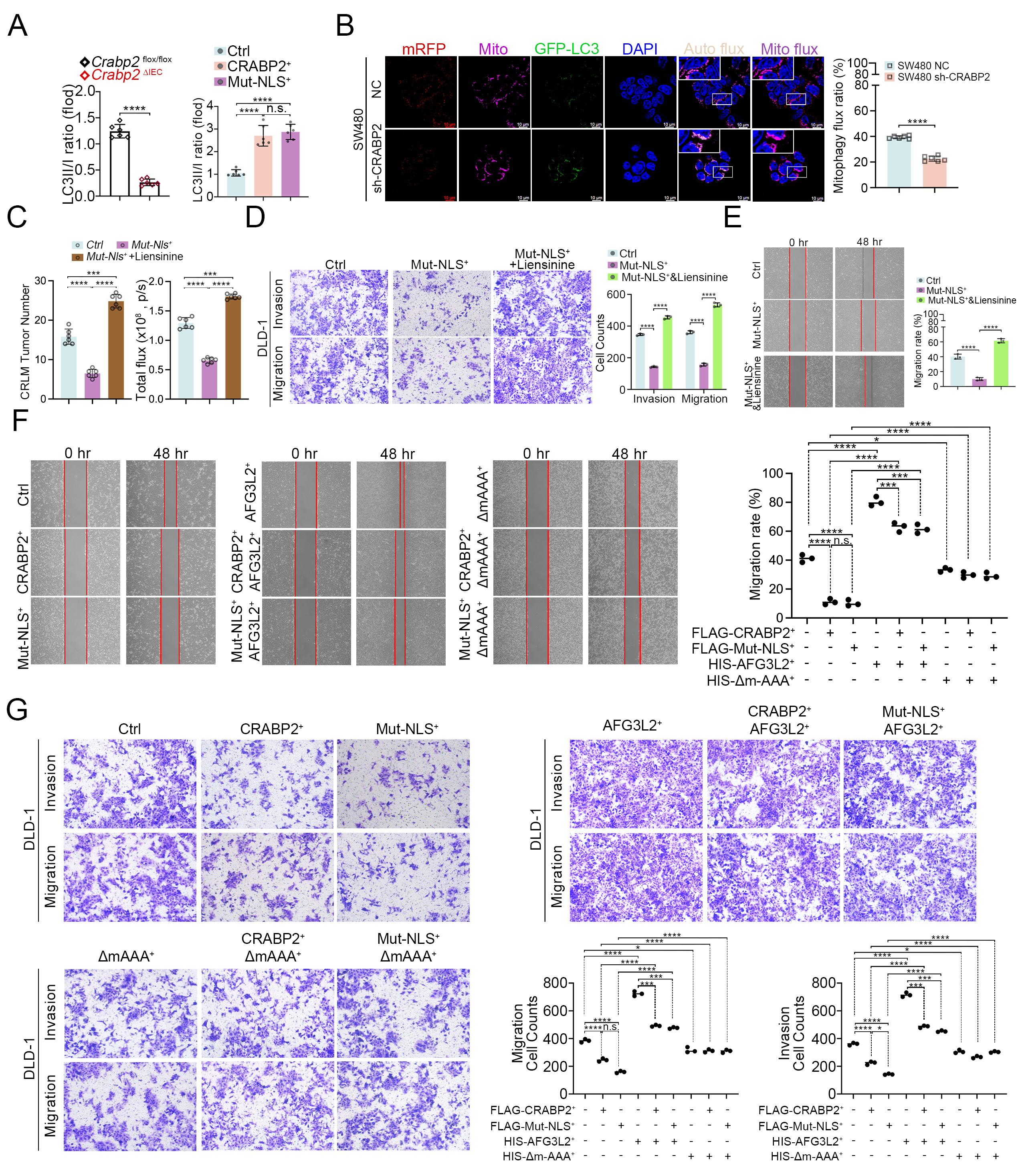
**

**Figure. S4.** Cytoplasmic CRABP2 Suppresses CRLM via Interacting with m-AAA Domain of AFG3L2 to Enhance PINK1-mediated Mitophagy. **(A)** The LC3II/I ratio described in **Fig. 4B** was quantitated. (**B**) Mitophagic flux assay in the mitophagy in the SW480 cells with knockdown CRABP2. (**C**) The quantity of CRLM and related luminescence intensity described in **Fig. 4E, F** was shown. (**D**) Transwell assays were used to detect the migration and invasion of CRC cells overexpressing Mut-NLS treated with vehicle/Liensinine. (**E**) Wound healing assays were used to detect the migration of CRC cells overexpressing Mut-NLS treated with vehicle/Liensinine. (**F**) Wound healing assays were used to detect the migration of DLD-1 cells overexpressing Flag-CRABP2, Flag-Mut-NLS with/without overexpressing HIS-AFG3L2 and HIS-AFG3L2 (Δm-AAA). (**G**) Transwell assays were used to detect the migration and invasion of DLD-1 cells overexpressing Flag-CRABP2, Flag-Mut-NLS with/without overexpressing HIS-AFG3L2 and HIS-AFG3L2 (Δm-AAA).

Original magnification ×63 (**B**), Bar=10 μm. The data are presented as the mean ± SD. n.s. no significant, ****p <0.0001, as analyzed by t-test (**A-B**) for comparisons between two groups, and by ANOVA followed by Tukey's honestly significant difference test (**C-G**) for comparisons between multiple groups.

**
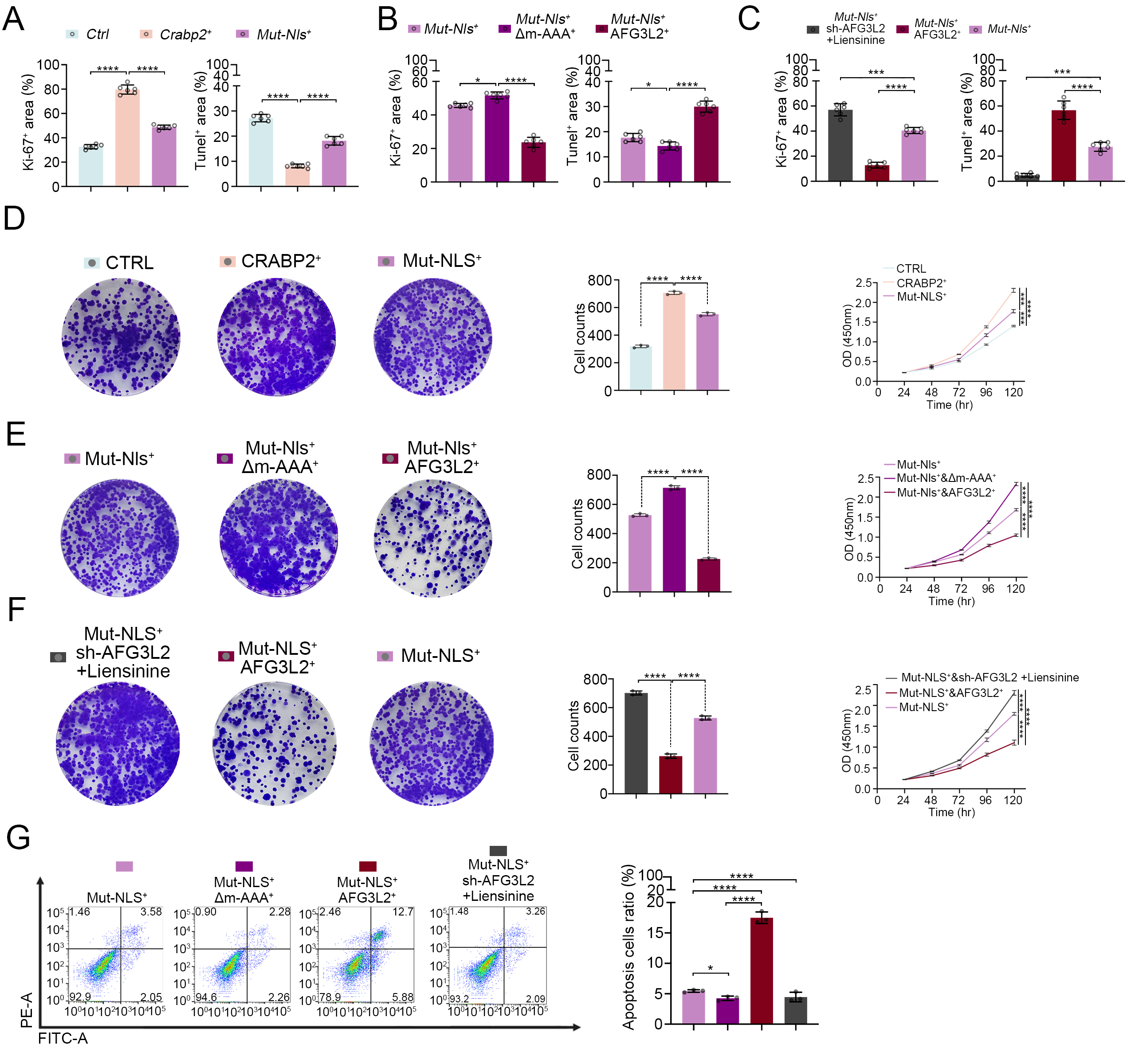
**

**Figure. S5.** AFG3L2-SLC25A39 Axis as a Mechanism for CRABP2-Mediated CRC Progression in the Cytoplasm. (**A**) The quantitation of immunohistochemistry (anti-Ki-67) and Tunel staining described in **Fig. 6B**. (**B**) The quantitation of immunohistochemistry (anti-Ki-67) and Tunel staining described in **Fig. 6D**. (**C**) The quantitation of immunohistochemistry (anti-Ki-67) and Tunel staining described in **Fig. 6F**. (**D**) Statistical results of plate colony in CRC cells with CRABP2 overexpression, Mut-NLS overexpression and Ctrl and CCK-8 assay was performed to determine the proliferation rate of CRC cells with CRABP2 overexpression, Mut-NLS overexpression and Ctrl. (**E**) Statistical results of plate colony in CRC cells with Mut-NLS overexpression, Mut-NLS/AFG3L2 double overexpression and Mut-NLS/ AFG3L2 (Δm-AAA) double overexpression and CCK-8 assay was performed to determine the proliferation rate of CRC cells with Mut-NLS overexpression, Mut-NLS/AFG3L2 double overexpression and Mut-NLS/ AFG3L2 (Δm-AAA) double overexpression. (**F**) Statistical results of plate colony and CCK-8 assay in CRC cells with Mut-NLS overexpression, Mut-NLS/AFG3L2 double overexpression and Mut-NLS overexpression&AFG3L2 knockdown treated with Liensinine and CCK-8 assay was performed to determine the proliferation rate of CRC cells with Mut-NLS overexpression, Mut-NLS/AFG3L2 double overexpression and Mut-NLS overexpression&AFG3L2 knockdown treated with Liensinine. (**G**) The flow cytometry (FCM) analysis of apoptosis in CRC cells with Mut-NLS overexpression, Mut-NLS/ AFG3L2 (Δm-AAA) double overexpression, Mut-NLS/AFG3L2 double overexpression and Mut-NLS overexpression&AFG3L2 knockdown treated with Liensinine. The data are presented as the mean ± SD. n.s. no significant, *p <0.05, ***p <0.001, ****p <0.0001, as analyzed by ANOVA and Tukey’s honestly significant difference (**A, B, C, D, E, F, G**).


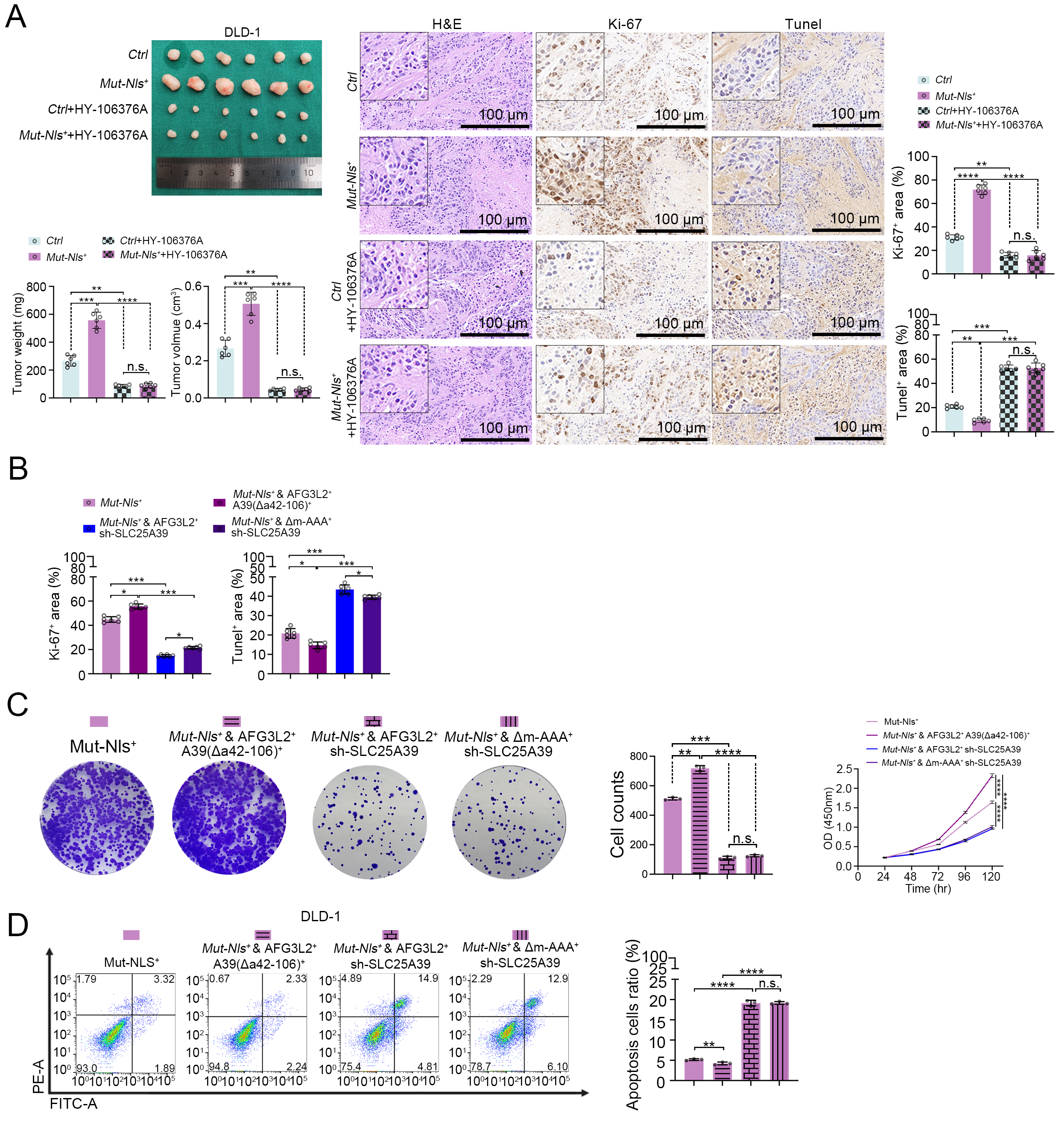


**Figure. S6.** AFG3L2-SLC25A39 Axis as a Mechanism for CRABP2-Mediated CRC Progression in the Cytoplasm.

(**A**) Subcutaneous tumorigenesis assays in nude mice injected with *CRABP2-Mut-NLS^+^*, or *Ctrl* cells treated with/without HY-106376A. Tumor weight and volume, H&E staining, immunohistochemical staining for Ki-67, and TUNEL staining are shown (*n* = 6)

(**B**) The quantitation of immunohistochemistry (anti-Ki-67) and Tunel staining described in **Fig. 6K**. (**C**) Statistical results of plate colony in Mut-Nls^+^ cells, Mut-Nls^+^&AFG3l2^+^& SLC25A39(Δ42-106)^+^ cells, Mut-Nls^+^&AFG3l2^+^&sh-SLC25A39 cells, and Mut-Nls^+^&AFG3L2(Δm-AAA)^+^&sh-SLC25A39 cells and CCK-8 assay was performed to determine the proliferation rate of Mut-Nls^+^ cells, Mut-Nls^+^&AFG3l2^+^& SLC25A39(Δ42-106)^+^ cells, Mut-Nls^+^&AFG3l2^+^&sh-SLC25A39 cells, and Mut-Nls^+^&AFG3L2(Δm-AAA)^+^&sh-SLC25A39 cells. (**D**) The flow cytometry (FCM) analysis of apoptosis in Mut-Nls^+^ cells, Mut-Nls^+^&AFG3l2^+^& SLC25A39(Δ42-106)^+^ cells, Mut-Nls^+^&AFG3l2^+^&sh-SLC25A39 cells, and Mut-Nls^+^&AFG3L2(Δm-AAA)^+^&sh-SLC25A39 cells. Original magnification ×40 (**A**), scale bar = 10 μm. The data are presented as the mean ± SD. n.s. no significant, *p <0.05, **p <0.01, ***p <0.001, ****p <0.0001, as analyzed by ANOVA and Tukey’s honestly significant difference (**A, B, C, D**).


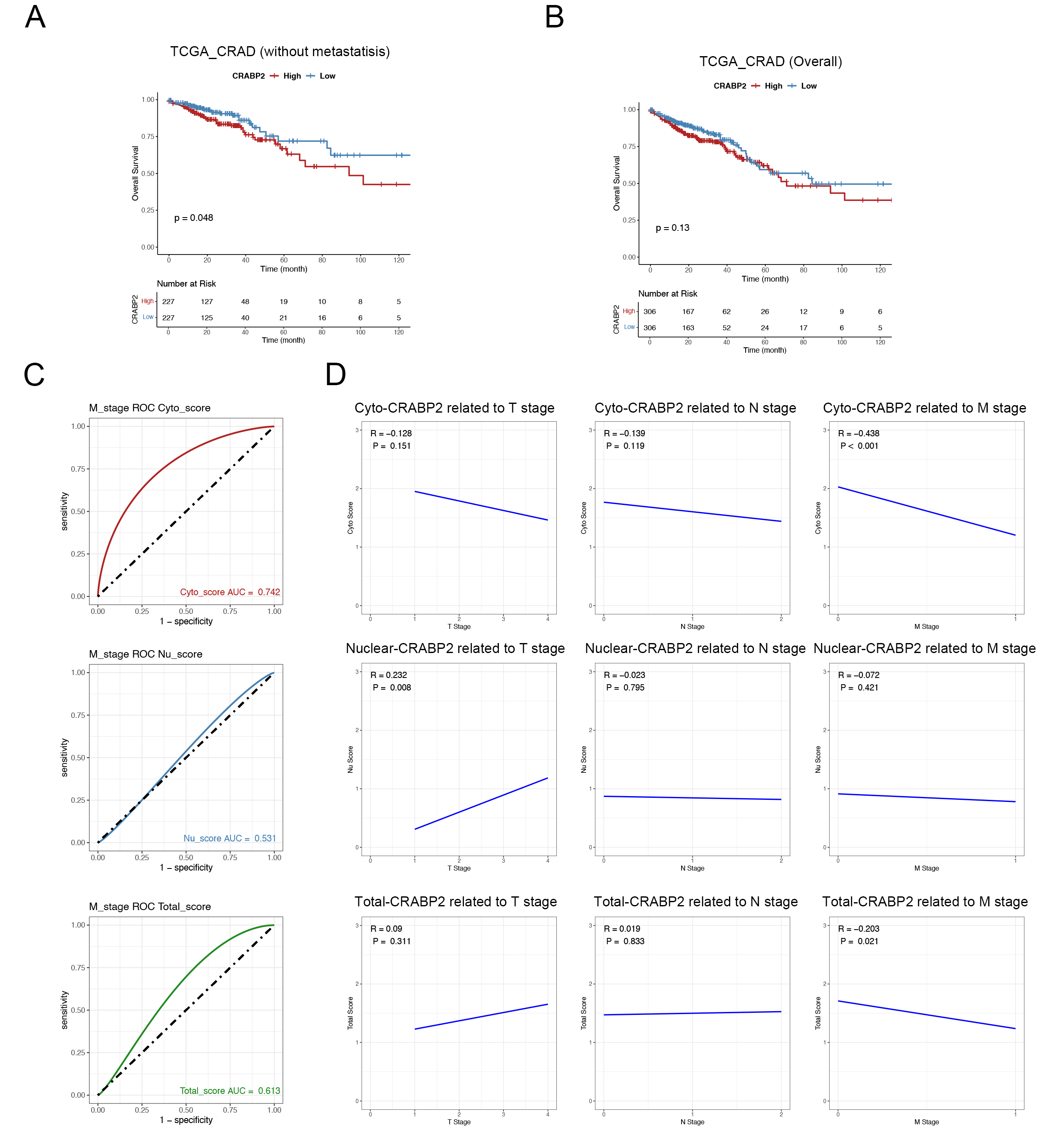


**Figure. S7.** CRABP2 was Significantly Upregulated in Colorectal Cancer and Its Localization had Distinct Prognostic in the Progression and Metastasis of Colorectal Cancer. (**A**) Kaplan-Meier survival curve for TCGA-COADREAD patients without metastasis, showing a significant difference in overall survival between high and low CRABP2 expression. (**B**) Kaplan-Meier survival curve for the overall TCGA-COADREAD cohort, showing no significant difference in overall survival between CRABP2 high and low groups. (**C**) ROC curves for cytoplasmic (Cyto), nuclear (Nu), and total CRABP2 scores predicting M-stage in 128 tissue microarray samples. (**D**) Scatter plots showing correlations between CRABP2 expression (cytoplasmic, nuclear, and total) and TNM stages in 128 tissue microarray samples. The p value of KM survival curves was calculated by the log-rank test.

**Table S1.** AOM-DSS induced CRC model in *Crabp2*^ΔIEC^ and *Crabp2*^flox/flox^ mice related to Fig. 1A.

| Parameter | *Crabp2*^flox/flox^ | *Crabp2*^ΔIEC^ |
| --- | --- | --- |
| Gender | Male | Male |
| Count | 6 | 6 |
| Length (cm) | 6.87 ± 0.19 | 8.01 ± 0.14^****^ |
| Number <2mm | 5.00 ± 1.26 | 5.33 ± 0.82 ^n.s.^ |
| Number >2mm | 6.83 ± 1.47 | 3.33 ± 0.82^****^ |
| Body Weight(g) | 26.78 ± 0.65 | 28.00 ± 0.78^*^ |
| Ki-67^+^ area (%) | 42.67 ± 3.14 | 14.50 ± 2.74^****^ |
| Tunel^+^ area (%) | 9.67 ± 2.16 | 23.33 ± 2.58^****^ |

The data are presented as the mean ± SD. n.s. no significant, * *p* <0.05, **** *p* <0.0001, as analyzed by t-test.

**Table S2.** AOM-DSS induced CRC model in *Crabp2*^ΔIEC^, *Crabp2*^flox/flox^ mice and *Crabp2*^ΔIEC^ tail injected with AAV9-sh-rb1 related to Fig. 2E.

| Parameter | *Crabp2*^flox/flox^ | *Crabp2*^ΔIEC^ | *Crabp2*^ΔIEC^&AAV9-sh-rb1 |
| --- | --- | --- | --- |
| Gender | Male | Male | Male |
| Count | 6 | 6 | 6 |
| Length (cm) | 7.00 ± 0.14 | 8.11 ± 0.19^****^ | 6.97 ± 0.15^####^ |
| Number <2mm | 5.00 ± 1.26 | 4.83 ± 0.75 ^n.s.^ | 5.17 ± 1.17^n.s.^ |
| Number >2mm | 7.50 ± 1.38 | 4.00 ± 0.89^***^ | 7.17 ± 1.17^###^ |
| Body Weight(g) | 26.75 ± 0.55 | 28.00 ± 0.52^**^ | 26.73 ± 0.34^###^ |
| Ki-67 | 36.83 ± 3.19 | 12.50 ± 1.87^****^ | 33.83 ± 2.32^####^ |
| Tunel | 14.00 ± 1.79 | 30.83 ± 4.12^****^ | 15.00 ± 2.90^####^ |

The data are presented as the mean ± SD.

n.s. no significant, ** *p* <0.01,*** *p* <0.001 *****p* <0.0001 (Ctrl vs cKO),

^###^ *p* <0.001 ^####^ *p* <0.0001 (cKO vs Rescue), as analyzed by t-test.

**Table S3.** The related characteristics of colon tumor tissue samples of human subjects with Cyto-CRABP.

| Cyto-CRABP Score | Score_0 | Score_1 | Score_2 | Score_3 |
| --- | --- | --- | --- | --- |
| Clinical_factor |  |  |  |  |
| Age_group |  |  |  |  |
| <65 | 6 | 23 | 20 | 14 |
| ≥65 | 7 | 25 | 18 | 15 |
| Sex |  |  |  |  |
| female | 3 | 17 | 13 | 11 |
| male | 10 | 31 | 25 | 18 |
| T_stage |  |  |  |  |
| 1 | 3 | 2 | 0 | 0 |
| 2 | 8 | 11 | 11 | 0 |
| 3 | 30 | 21 | 11 | 9 |
| 4 | 7 | 4 | 7 | 4 |
| N_stage |  |  |  |  |
| 0 | 5 | 23 | 15 | 20 |
| 1 | 5 | 14 | 12 | 6 |
| 2 | 3 | 11 | 11 | 3 |
| M_stage |  |  |  |  |
| 0 | 2 | 18 | 25 | 24 |
| 1 | 11 | 30 | 13 | 5 |

**Table S4.** The related characteristics of colon tumor tissue samples of human subjects with Nu-CRABP.

| Nu-CRABP Sccore | Score_0 | Score_1 | Score_2 | Score_3 |
| --- | --- | --- | --- | --- |
| Clinical_factor |  |  |  |  |
| Age_group |  |  |  |  |
| <65 | 25 | 25 | 7 | 6 |
| ≥65 | 31 | 20 | 10 | 4 |
| Sex |  |  |  |  |
| female | 21 | 14 | 4 | 5 |
| male | 35 | 31 | 13 | 5 |
| T_stage |  |  |  |  |
| 1 | 4 | 1 | 0 | 0 |
| 2 | 16 | 10 | 3 | 1 |
| 3 | 30 | 27 | 6 | 8 |
| 4 | 6 | 7 | 8 | 1 |
| N_stage |  |  |  |  |
| 0 | 28 | 19 | 10 | 6 |
| 1 | 17 | 15 | 3 | 2 |
| 2 | 11 | 11 | 4 | 2 |
| M_stage |  |  |  |  |
| 0 | 30 | 21 | 12 | 6 |
| 1 | 26 | 24 | 5 | 4 |

**Table S5.** The related characteristics of colon tumor tissue samples of human subjects with Total-CRABP.

| Total-CRABP Sccore | Score_0 | Score_1 | Score_2 | Score_3 |
| --- | --- | --- | --- | --- |
| Clinical_factor |  |  |  |  |
| Age_group |  |  |  |  |
| <65 | 13 | 18 | 12 | 20 |
| ≥65 | 21 | 15 | 13 | 16 |
| Sex |  |  |  |  |
| female | 13 | 14 | 4 | 13 |
| male | 21 | 19 | 21 | 23 |
| T_stage |  |  |  |  |
| 1 | 2 | 3 | 0 | 0 |
| 2 | 10 | 8 | 6 | 6 |
| 3 | 18 | 15 | 24 | 14 |
| 4 | 4 | 10 | 3 | 5 |
| N_stage |  |  |  |  |
| 0 | 18 | 14 | 11 | 20 |
| 1 | 12 | 8 | 10 | 7 |
| 2 | 4 | 11 | 4 | 9 |
| M_stage |  |  |  |  |
| 0 | 15 | 16 | 12 | 26 |
| 1 | 19 | 17 | 13 | 10 |

**Table S6.** The related materials used in research.

| **Antibody** | | | | | | | | | | | | | |
| --- | --- | --- | --- | --- | --- | --- | --- | --- | --- | --- | --- | --- | --- |
| **Name** | | | **Citation** | | | | **Supplier** | | | | **Cat no.** | | **Clone no.** |
| Rabbit Polyclonal anti-CRABP2 | | | Kaiho-Soma A, et al. *Mol Cell*. 2021 Apr 1;81(7):1411-1424.e7. | | | | Proteintech | | | | 10225-1-AP | | N/A |
| Rabbit Polyclonal anti-CRABP1 | | | Zhu Y, et al. *Elife*. 2019 Nov 4;8:e50777. | | | | Proteintech | | | | 12588-1-AP | | N/A |
| Rabbit IgG control Polyclonal antibody | | | Wei D, et al. *Cell Res*. 2021 Feb;31(2):157-177. | | | | Proteintech | | | | 30000-0-AP | | N/A |
| Rabbit Polyclonal anti-AFG3L2 | | | Tsai CW, et al. *Mol Cell*. 2022 Oct 6;82(19):3661-3676.e8. | | | | Proteintech | | | | 14631-1-AP | | N/A |
| Mouse Polyclonal anti-AFG3L2 | | | Jiang X, et al. *Proc Natl Acad Sci U S A*. 2014 Oct 14;111(41):14782-7. | | | | Abcam | | | | ab68023 | | N/A |
| Rabbit Polyclonal anti-RB1 | | | Yuan K, et al. *Nat Commun*. 2022 May 25;13(1):2903. | | | | Proteintech | | | | 10048-2-Ig | | N/A |
| Mouse Monoclonal anti-RB1 | | | N/A | | | | Proteintech | | | | 67521-1-Ig | | 1A2A6 |
| Rabbit monoclonal Anti-SLC25A39 | | | N/A | | | | Abcam | | | | ab315170 | | EPR28609-42 |
| LC3 Polyclonal antibody | | | Wang D, et al. *Gastroenterology*. 2023 Dec;165(6):1488-1504.e20. | | | | Proteintech | | | | 14600-1-AP | | N/A |
| Rabbit monoclonal Anti-PINK1 | | | Nan D, et al. *Nat Commun*. 2024 Jun 4;15(1):4740. | | | | Cell Signaling Technology | | | | 6946 | | D8G3 |
| Mouse Monoclonal anti-Alpha Tubulin | | | Chen L, et al. *Nature*. 2023 Jun;618(7966):862-870. | | | | Proteintech | | | | 66031-1-Ig | | 1E4C11 |
| Mouse Monoclonal anti-Lamin B1 | | | Shen H, et al. *Nat Commun*. 2022 Oct 13;13(1):6030. | | | | Proteintech | | | | 66095-1-Ig | | 3C10G12 |
| Mouse Monoclonal anti-COXIV | | | Wang Q, et al. *Science*. 2021 Sep 17;373(6561):1377-1381. | | | | Proteintech | | | | 66110-1-Ig | | N/A |
| Rabbit monoclonal anti-TOMM20 | | | Bond KH, et al. *BMC Cancer*. 2021;21(1):312. | | | | Abcam | | | | ab186735 | | EPR15581-54 |
| Rabbit polyclonal anti-Tim23 | | | Hoshino A, et al. *Nature*. 2019;575(7782):375-379. | | | | Proteintech | | | | 11123-1-AP | | N/A |
| Rabbit monoclonal anti-SQSTM1/p62 | | | Li W, et al. *Autophagy*. 2021;17(6):1410-1425. | | | | Abcam | | | | ab109012 | | EPR4844 |
| Rabbit monoclonal  anti-FLAG | | | Zhou Y, et al. *Signal Transduct Target Ther*. 2024 Jun 28;9(1):159. | | | | Cell Signaling Technology | | | | 14793 | | D6W5B |
| Rabbit monoclonal  anti-His-Tag | | | Liu H, et al. *Cell Death* Discov. 2022;8(1):403. | | | | Cell Signaling Technology | | | | 12698 | | D3I1O |
| Rabbit monoclonal  anti-HA-Tag | | | Huoh YS, et al. *Nat Immunol*. 2024 Sep;25(9):1580-1592. | | | | Cell Signaling Technology | | | | 3724 | | C29F4 |
| Anti-rabbit IgG, HRP-linked Antibody | | | Zhang W, et al. *Immunity*. 2021;54(6):1168-1185. | | | | Cell Signaling Technology | | | | 7074 | | N/A |
| Anti-mouse IgG, HRP-linked Antibody | | | Zhang W, et al. *Immunity*. 2021;54(6):1168-1185. | | | | Cell Signaling Technology | | | | 7076 | | N/A |
|  | | | | | | | | | | | | | |
| **Cell lines** | | | | | | | | | | | | | |
| **Name** | **Citation** | | | | **Supplier** | | | **Cat no.** | | | | **Authentication test method** | |
| DLD-1 | Witty JP, et al. *Cancer Res*. 1994 Sep 1;54(17):4805-12. | | | | Cell Bank of Chinese Science Academy | | | SCSP-5241 | | | | STR | |
| SW480 | Ghandi M, et al. *Nature*. 2019 May;569(7757):503-508. | | | | Cell Bank of Chinese Science Academy | | | SCSP-5033 | | | | STR | |
| HEK293T | Zhang W, et al. *Immunity*. 2021;54(6):1168-1185. | | | | Cell Bank of Chinese Science Academy | | | SCSP-502 | | | | STR | |
| MC38 | Tan MH, et al. *J Natl Cancer Inst*. 1976 Apr;56(4):871-3. | | | | Cell Bank of Chinese Science Academy | | | SCSP-5431 | | | | STR | |
|  | | | | | | | | | | | | | |
| **Organisms** | | | | | | | | | | | | | |
| **Name** | | **Supplier** | | | | **Strain** | | **Sex** | | **Age** | | | |
| *Crabp2*^flox/flox^ | | Cyagen | | | | C57BL/6 | | Male | | 6 or 8 weeks old | | | |
| *Vil1-Cre transgenic* | | Cyagen | | | | C57BL/6 | | Male | | 6 or 8 weeks old | | | |
| *Wildtype C57/Bl6* | | GemPharmatech Co., Ltd. | | | | C57BL/6 | | Male | | 6 or 8 weeks old | | | |
| *Nude mice* | | GemPharmatech Co., Ltd. | | | | *Nude mice* | | Male | | 6 or 8 weeks old | | | |
|  | | | | | | | | | | | | | |
| **Sequence based reagents** | | | | | | | | | | | | | |
| **Name** | | | | **Sequence** | | | | | **Supplier** | | | | |
| Ho-CRABP2 | | | | Forward: ATCGGAAAACTTCGAGGAATTGC Reverse:  AGGCTCTTACAGGGCCTCC | | | | | Generay Biotechnology | | | | |
| Ho-AFG3L2 | | | | Forward:  TCCCAAAGGGTGCCATTCTC Reverse:  TCTTCCGAGCAAGGGCAAAT | | | | | Generay Biotechnology | | | | |
| Ho-RB1 | | | | Forward:  GAGTCGGGAGAGGACGGG Reverse: TTCAAACTCAAGCCTGACGAGA | | | | | Generay Biotechnology | | | | |
| Ho-SLC25A39 | | | | Forward:  CTGGAGCTTATGCGGACAAA Reverse:  GAAGGGCACATCTCGAAGG | | | | | Generay Biotechnology | | | | |
| *Mus-Crabp2* | | | | Forward:  GGGTCTACGTCCGAGAGTGA Reverse:  GTGGGAGGGAGGTTTGTGTC | | | | | Generay Biotechnology | | | | |
| *Mus-Rb1* | | | | Forward:  TCTCACCTCCTGCACTACTCA Reverse:  TGACCTCTTCTGGGTGTTCG | | | | | Generay Biotechnology | | | | |
| *Mus-gapdh* | | | | Forward:  GGAGAGTGTTTCCTCGTCCC Reverse:  GATGGGCTTCCCGTTGATGA | | | | | Generay Biotechnology | | | | |
| *Mus-Crabp2 loxp Primers1* | | | | Forward:  AGTGGGCATGGGGAATTTAGTAAC Reverse:  ATTCCAGCAACACATTCCTAGCAC | | | | | Generay Biotechnology | | | | |
| *Vil1-Cre* | | | | Forward:  CCATAGGAAGCCAGTTTCCCTTC  Reverse: TTCCAGGTATGCTCAGAAAACGC | | | | | Generay Biotechnology | | | | |
|  | | | | | | | | | | | | | |
| **Biological samples** | | | | | | | | | | | | | |
| **Description** | | | | **Source** | | | | | **Identifier** | | | | |
| Human Colon tumor and adjacent tissues | | | | Supplied by Dr. Yueming Sun | | | | | The First Affiliated Hospital of Nanjing Medical University (2022-SRFA-142) | | | | |
|  | | | | | | | | | | | | | |
| **Deposited data** | | | | | | | | | | | | | |
| **Name of repository** | | | | **Identifier** | | | | | **Link** | | | | |
| Human CRC cells raw mass spectrometry data | | | | This paper | | | | | N/A | | | | |
|  | | | | | | | | | | | | | |
| **Software** | | | | | | | | | | | | | |
| **Software name** | | | | **Manufacturer** | | | | | **Version** | | | | |
| GraphPad Prism 10.0 | | | | GraphPad Software | | | | | La Jolla, CA, USA | | | | |
| SPSS 25.0 | | | | SPSS Software | | | | | Chicago, IL, USA | | | | |
| Origin Lab software | | | | OriginLab | | | | | N/A | | | | |
| Photoshop 2024 | | | | Adobe | | | | | N/A | | | | |
| Image J | | | | NIH | | | | | N/A | | | | |
| ImageLab | | | | ImageLab | | | | | N/A | | | | |
| Leica Application Suite X | | | | Leica | | | | | N/A | | | | |
| FlowJo | | | | TreeStar | | | | | N/A | | | | |
| StepOne software v2.3 | | | | StepOne Software | | | | | N/A | | | | |
| CaseViewer | | | | CaseViewer Software | | | | | N/A | | | | |
| K-Viewer | | | | KFBIO | | | | | N/A | | | | |
|  | | | | | | | | | | | | | |
| **Others (e.g. drugs, proteins, vectors etc.)** | | | | | | | | | | | | | |
| Puromycin | | | | Sigma-Aldrich | | | | | Cat# P8833 | | | | |
| CCCP | | | | Sigma-Aldrich | | | | | Cat#C2759 | | | | |
| Liensinine | | | | Pureone Bio Technology | | | | | Cat#P0943 | | | | |
| L-Buthionine-(S,R)-sulfoximine | | | | MCE | | | | | Cat#HY-106376A | | | | |
| Electron microscope fixation fluid | | | | Servicebio | | | | | Cat #G1102 | | | | |
| The UltraSensitiveTM SP (Mouse/Rabbit) IHC Kit | | | | Maixin Biotech | | | | | Cat#KIT-9730 | | | | |
| TRIzol™ Reagent | | | | Invitrogen | | | | | Cat#15596018CN | | | | |
| Annexin V-FITC/PI Apoptosis Detection Kit | | | | Vazyme | | | | | Cat#A211 | | | | |
| HiScript III RT SuperMix for qPCR (+gDNA wiper) | | | | Vazyme | | | | | Cat# R323-01 | | | | |
| Goat anti-Rabbit IgG (H+L) Cross-Adsorbed Secondary Antibody, Alexa Fluor™ 647 | | | | Invitrogen | | | | | Cat# A-21244 | | | | |
| Goat anti-Mouse IgG (H+L) Cross-Adsorbed Secondary Antibody, Alexa Fluor™ 488 | | | | Invitrogen | | | | | Cat# A-11001 | | | | |
| Pierce™ Classical magnetic bead method IP/Co-IP Kit | | | | Thermo Scientific | | | | | Cat#88804 | | | | |
| Fast Silver Stain Kit | | | | Beyotime | | | | | Cat#P0017S | | | | |
| Azoxymethane | | | | Sigam-Aldrich | | | | | Cat#A5486 | | | | |
| Dextran Sulfate Sodium Salt | | | | MPBiomedicals | | | | | Cat#160110 | | | | |
| D-luciferin potassium salt | | | | GOLDBIO | | | | | Cat#115144-35-9 | | | | |
| Mitochondria Isolation Kit | | | | Beyotime | | | | | Cat#C3601 | | | | |
| PARIS™ Kit | | | | Invitrogen | | | | | Cat# AM1921 | | | | |
| BCA Protein Assay Kit | | | | Beyotime | | | | | Cat#P0012 | | | | |
| GSH and GSSG Assay Kit | | | | Beyotime | | | | | Cat#S0053 | | | | |
| Premo™ Autophagy Tandem Sensor mRFP-GFP-LC3 kit | | | | Invitrogen | | | | | Cat# P36239 | | | | |
| CCK-8 Cell Counting Kit | | | | Vazyme | | | | | Cat#A311 | | | | |
| ChamQ Universal SYBR® qPCR Master Mix | | | | Vazyme | | | | | Cat#Q711-02 | | | | |
| Lipofectamine™ 3000 Transfection Reagent | | | | Invitrogen | | | | | Cat# L3000150 | | | | |
| RPMI 1640 | | | | Gibco™ | | | | | Cat#11875093 | | | | |
| DMEM | | | | Gibco™ | | | | | Cat#11965092 | | | | |
| DMEM/F-12 | | | | Gibco™ | | | | | Cat#11320033 | | | | |
| Fetal Bovine Serum | | | | Gibco™ | | | | | Cat#A5669402 | | | | |
| Penicillin-Streptomycin | | | | Gibco™ | | | | | Cat#15140122 | | | | |
| PGMLV-CMV-MCS-PGK-Puro | | | | Genomeditech | | | | | N/A | | | | |
| Lentiviral vector pLKO.1-puro | | | | Genomeditech | | | | | N/A | | | | |
| Vector sequence: TTCTCCGAACGTGTCACGT | | | | Genomeditech | | | | | N/A | | | | |
| shRNA targeting sequence: Ho-CRABP2#1: CCACAGAGATTAACTTCAA | | | | Genomeditech | | | | | N/A | | | | |
| shRNA targeting sequence: Ho-CRABP2#2: GGGAACTGATCCTGACCATGA | | | | Genomeditech | | | | | N/A | | | | |
| shRNA targeting sequence: Ho-CRABP2#3: GGGTGAATGTGATGCTGAGGA | | | | Genomeditech | | | | | N/A | | | | |
| shRNA targeting sequence: Ho-AFG3L2#1: GGGCAATACGTTTGGTTTAAT | | | | Genomeditech | | | | | N/A | | | | |
| shRNA targeting sequence: Ho-AFG3L2#2: GCTAGAGTCCGAGACTTATTT | | | | Genomeditech | | | | | N/A | | | | |
| shRNA targeting sequence: Ho-AFG3L2#3: TTCGACAGGCAGATCTTTATT | | | | Genomeditech | | | | | N/A | | | | |
| AAV9-NC sequence: TTCTCCGAACGTGTCACGT | | | | Genomeditech | | | | | N/A | | | | |
| AAV9-Sh-rb1 sequence: CCGTGGATTCTGAACGTACTT | | | | Genomeditech | | | | | N/A | | | | |
| shRNA targeting sequence: Ho-SLC25A39#1: CCAAGTTCAAGACCAAATCTT | | | | Genomeditech | | | | | N/A | | | | |
| shRNA targeting sequence: Ho-SLC25A39#2: CTGGAGCTTATGCGGACAAAG | | | | Genomeditech | | | | | N/A | | | | |
| shRNA targeting sequence: Ho-SLC25A39#3: TCTACCCTTTGACGTGGTAAA | | | | Genomeditech | | | | | N/A | | | | |

**Please provide the details of the corresponding methods author for the manuscript:**

| Lead contact: Yueming Sun Address: Department of General Surgery, Colorectal Institute of Nanjing Medical University, The First Affiliated Hospital of Nanjing Medical University, Nanjing, China. 210029. TEL: 86-25-68306026 Fax: 86-25-68306026  Electronic mail: [sunyueming@njmu.edu.cn](mailto:sunyueming@njmu.edu.cn). |
| --- |
